# The impacts of COVID-19 on eating disorders and disordered eating: A mixed studies systematic review and implications

**DOI:** 10.3389/fpsyg.2022.926709

**Published:** 2022-09-06

**Authors:** Courtney P. McLean, Ranjani Utpala, Gemma Sharp

**Affiliations:** ^1^Monash Alfred Psychiatry Research Centre, Monash University, Melbourne, VIC, Australia; ^2^Butterfly Foundation, Sydney, NSW, Australia

**Keywords:** eating disorder, disordered eating, eating behavior and eating disorder, systematic review, COVID-19, pandemic (COVID-19)

## Abstract

**Purpose:**

The unique constraints to everyday life brought about by the COVID-19 pandemic have been suggested to negatively impact those with pre-existing mental health issues such as eating disorders. While individuals with eating disorders or disordered eating behaviors likely represent a vulnerable group to the COVID-19 pandemic, the impact of the pandemic is yet to be fully established.

**Methods:**

We systematically examined the impact of the COVID-19 pandemic on eating disorders and disordered eating behaviors. We searched electronic databases MEDLINE, PsycINFO, CINAHL, and EMBASE for literature published until October 2021. Eligible studies were required to report on individuals with or without a diagnosed eating disorder or disordered eating behaviors who were exposed to the COVID-19 pandemic.

**Findings:**

Seventy-two studies met eligibility criteria with the majority reporting an increase in eating disorder or disordered eating behaviors associated with the COVID-19 pandemic. Specifically, it appears children and adolescents and individuals with a diagnosed eating disorder may present vulnerable groups to the impacts of the COVID-19 pandemic.

**Discussion:**

This mixed systematic review provides a timely insight into COVID-19 eating disorder literature and will assist in understanding possible future long-term impacts of the pandemic on eating disorder behaviors. It appears that the role of stress in the development and maintenance of eating disorders may have been intensified to cope with the uncertainty of the COVID-19 pandemic. Future research is needed among understudied and minority groups and to examine the long-term implications of the COVID-19 pandemic on eating disorders and disordered eating behaviors.

**Systematic review registration:**

https://www.crd.york.ac.uk/prospero/display_record.php?RecordID=284749, PROSPERO [CRD42021284749].

## Introduction

The emergence of the coronavirus disease (COVID-19), leading to the COVID-19 pandemic, created unique constraints to everyday life. Across the world, governments attempted to contain the spread of the disease through home-confinement, closing of non-essential businesses, personal protective equipment (PPE) mandates, and nightly curfews. For many countries, these COVID-19 mitigation measures have sustained long-term, such as social-distancing and PPE wearing, to prevent transmission of the disease, even once vaccination campaigns have ceased (Jarvis et al., 2020; Reinders Folmer et al., 2021). These measures have been shown to negatively impact the mental health of the general population, but particularly adversely affect those with pre-existing mental health issues (Kumar and Nayar, 2021). A systematic review and meta-analysis on the prevalence of anxiety, depression, and psychological distress in the general population found a high prevalence of overall mental ill-health during the COVID-19 pandemic (Necho et al., 2021). This is further corroborated by reviews suggesting high prevalence rates of anxiety and depression in a diverse range of populations including healthcare workers (e.g., Li et al., 2021), university students (e.g., Wang C. et al., 2021), adolescents (e.g., Nearchou et al., 2020), and pregnant women (e.g., Sun et al., 2021). As such, the impact of the COVID-19 pandemic on mental health both short- and long-term remains an urgent public health and research priority.

Individuals with eating disorders or disordered eating behaviors likely represent a vulnerable group for which the COVID-19 pandemic impact is yet to be fully established. Eating disorders are estimated to affect up to 9% of the population worldwide and, taken together, are considered to be one of the most fatal of all mental illnesses (Arcelus et al., 2011). In Australia, the impact of COVID-19 on eating disorders has been supported by the Butterfly Foundation's National Helpline noting a 116% increase in contacts since the beginning of the pandemic (Butterfly Foundation, 2020), with similar reports from the United Kingdom's eating disorder charity, Beat (2021). This has also been supported by preliminary qualitative research in patients with anorexia nervosa finding they experienced heightened psychological distress and increased attempts to self-manage their recovery during this time (Clark Bryan et al., 2020). It is thought that psychosocial stressors related to a disruption in daily routine, social distancing restrictions, stay at home orders, and limited access to specific foods creating food insecurity can be extremely distressing to individuals with an existing eating disorder (Gordon and Katzman, 2020; Phillipou et al., 2020). These stressors can also be extended to those in the general population by increasing risk and vulnerability to disordered eating behaviors such as binge eating and food restriction (Phillipou et al., 2020; Brownstone et al., 2021).

While the literature suggests the COVID-19 pandemic has negatively affected individuals with eating disorders and disorder eating behaviors, the extent of the impact is unknown. A limited number of published systematic reviews exist on the topic of COVID-19 and eating disorders, though they are generally driven by specific inclusion and exclusion criteria. For example, they include studies examining specific eating disorders such as anorexia nervosa and exclude studies examining participants with disordered eating behaviors (Dumitracu et al., 2021; Miniati et al., 2021; Sideli et al., 2021). As disordered eating behaviors are known to be disproportionally widespread in the community (Reba-Harrelson et al., 2009) and are a known risk factor for the development of eating disorders, it remains important to establish the impact of the COVID-19 pandemic more broadly in individuals who do not meet criteria for an eating disorder. To our knowledge, only one scoping review has investigated the relationship between eating disorders, disordered eating behaviors, and the COVID-19 pandemic (Linardon et al., 2022). Our mixed studies systematic review represents an extension to Linardon et al. (2022)'s work by including studies of qualitative nature, as these present important additions to the field that focus on meanings and interpretations of the participants. In doing so, we aim to reduce publication bias bought about by qualitative studies without distinct or easily described findings less likely to be published in peer-reviewed journals (Petticrew et al., 2008). Taken together with the limitations of previous literature, an all-encompassing mixed studies systematic review examining both eating disorders in clinical populations and disordered eating behaviors (e.g., binge eating behaviors, dietary restriction; Pereira and Alvarenga, 2007) in the community in relation to the COVID-19 pandemic is crucial. Through this process we can begin to understand the potential long-term health impacts of the COVID-19 pandemic, including key literature gaps and future research needs, in turn informing the strategies and policies of governments in generating additional investments into the mental health space.

The aim of this study is to conduct a mixed studies systematic review to examine the manifestations of the COVID-19 pandemic on eating disorders and disordered eating behaviors. Our mixed systematic review aims to present a comprehensive investigation into pathological eating behaviors in individuals with and without a diagnosed eating disorder across the age spectrum. Furthermore, this mixed systematic review will pay particular attention to understudied and minority groups such as children and adolescents, geriatric populations, people from culturally and linguistically diverse backgrounds, LGBTIQA+ community, Indigenous peoples, and the gender spectrum. Such groups are consistently underrepresented among eating disorder literature but are also known to be less likely to receive a diagnosis and face greater challenges accessing treatment (Sim, 2019; Brownstone et al., 2021). For this reason, it is important to collect literature to better understand how to support these groups during the COVID-19 pandemic and beyond. Lastly, we will provide a critical contribution to the field by making recommendations on the impact of the COVID-19 pandemic and eating disorders from a self, carer, and healthcare professional outlook.

## Methods

This review comprises a mixed studies systematic review which integrates qualitative, quantitative, and mixed methods studies. This mixed studies systematic review was conducted in accordance with the Preferred Reporting Items for Systematic Reviews and Meta-Analysis (PRISMA) statement (Moher et al., 2009). The study was registered on PROSPERO on 15th October 2021 (registration number CRD42021284749) and our methods did not deviate from the registration.

### Search strategy

Relevant literature was identified through a manual search of electronic bibliographic databases MEDLINE, PsycINFO, CINAHL, and EMBASE on the 8th October 2021. Search and MeSH terms appearing in either title, abstract, subject heading, or keyword were used, including: (“eating disorder^*^” OR “disordered eating” OR anorexi^*^ OR “anorexia nervosa” OR bulimi^*^ OR “bulimia nervosa” OR orthorexi^*^ OR “orthorexia nervosa” OR “binge eating disorder” OR “binge eat^*^” OR “restricted eating” OR “feeding disorder^*^” OR “cognitive restraint” OR “cognitive dietary restraint” OR “cognitive eating restraint” OR “restricted eating” OR “restricted food intake disorder” OR “rumination disorder” OR “food intake disorder^*^” OR “purging disorder^*^” OR “other specified feeding” OR “unspecified feeding” OR pica) AND (coronavirus OR corona OR COVID-19 OR COVID19 OR COVID OR SARS-CoV-2 OR SARSCoV-2 OR “novel coronavirus” OR “SARS virus” OR pandemic OR “severe acute respiratory syndrome”).

### Inclusion criteria

Inclusion criteria followed the PICOS formatting (Participant, Intervention, Comparison, Outcomes, Study Design) (Liberati et al., 2009), with studies required to meet the following criteria to be eligible for inclusion.

#### Participants

Studies on individuals of any age or gender, with a diagnosed eating disorder or disordered eating behaviors, or without (e.g., carers of patients with an eating disorder).

#### Interventions

Studies on individuals exposed to the COVID-19 pandemic.

#### Comparisons

Studies that reported eating disorder or disordered eating behaviors relative to pre-COVID-19 pandemic times or none.

#### Outcomes

Studies assessing eating disorders or disordered eating behaviors associated with the COVID-19 pandemic. Eating disorders may include the onset, or worsening of, symptoms associated with a diagnosed eating disorder including anorexia nervosa, bulimia nervosa, binge eating disorder, avoidant restrictive food intake disorder, rumination disorder, PICA, or other specified feeding and eating disorder. Disordered eating behaviors may include the onset, or worsening of, any form of pathological eating behaviors or attitudes such as binge eating, food restriction, excessive exercise, purging, or other methods to control or limit weight, that do not reach a clinical diagnosis (Pereira and Alvarenga, 2007). Specifically, disordered eating behaviors can be considered a multidimensional construct with a core function of the behavior to alleviate stress caused by body image concerns (Pereira and Alvarenga, 2007).

#### Studies

Commentaries, editorial, letters, perspectives, systematic reviews, meta-analyses, narrative reviews, protocols, animal studies, case studies, abstracts, and conference posters were excluded. Dissertations published through ProQuest Dissertations Publishing were allowed. Publication date was restricted to December 2019 and beyond to capture the emergence of the COVID-19 outbreak. No restrictions to data collection dates were applied.

### Exclusion criteria

Studies were restricted to those published in English language. We wanted to focus on behaviors known to be associated with eating disorders, therefore studies were excluded if they reported on general eating habits and related behaviors, such as increased snacking or exercise not related to weight control or body image concerns in relation to the COVID-19 pandemic.

### Study selection

First review author, CM, screened identified studies against inclusion and exclusion criteria using systematic review management platform, Covidence. Studies were screened hierarchically with titles and abstracts of searched literature screened first, followed by full text to identify eligible studies. Eligible studies were imported to database management software, Endnote, for data extraction. If multiple articles for a single study were available, the most recent publication date was used. Senior review author, GS, independently reviewed 5% of full text articles pre-data extraction to ensure accuracy against eligibility criteria, and an additional 5% post-data extraction to confirm inclusion of studies (Liberati et al., 2009). If discrepancies did arise during the bias assessment, disagreements were resolved by consensus.

### Data extraction and synthesis

Study characteristics and findings were extracted and synthesized using tables. Study characteristics and findings include author name(s) and date of publication, country of origin, study design, sample characteristics including study population, sample sizes, clinical status, ethnicity/race, gender and age distribution, socio-economic status, belonging to an understudied or minority group, tool or measure used to assess eating disorder or disordered eating behaviors, main findings, and Mixed Methods Appraisal Tool rating. For qualitative studies, the main themes reported by each study, also known as second-order constructs (Toye et al., 2014), were extracted and, respectively, listed under Main Findings in Supplementary Table 1. Due to large heterogeneity between study samples (e.g., *N* = 10–12,186), study design (e.g., cross-sectional, longitudinal, retrospective analysis), and measures (e.g., EDE, EDE-Q, CIA, DSM-5) (Esterhuizen and Thabane, 2016), we elected not to pool data into a statistical meta-analysis due to concerns for publications bias and skewed data. Therefore, we provide a grouping synthesis by population and study design.

### Quality assessment

The methodological quality of studies was assessed using the Mixed Methods Appraisal Tool (MMAT; Hong et al., 2018). The MMAT allows authors to critically assess methodological quality in both quantitative, qualitative, and mixed methods studies against five categories. While MMAT authors discourage calculating an overall numerical score from the ratings, a ranking system has been developed to convey such results (Wong et al., 2020). A study is classified as high if all five categories are met, medium if four categories are met, and low if three or less categories are met (see Supplementary Information for study design categories; Wong et al., 2020). First review author, CM, conducted the initial quality assessment and senior review author, GS, independently reviewed 5% of assessments. Disagreements were resolved by consensus.

## Results

### Study selection

**Figure 1:**
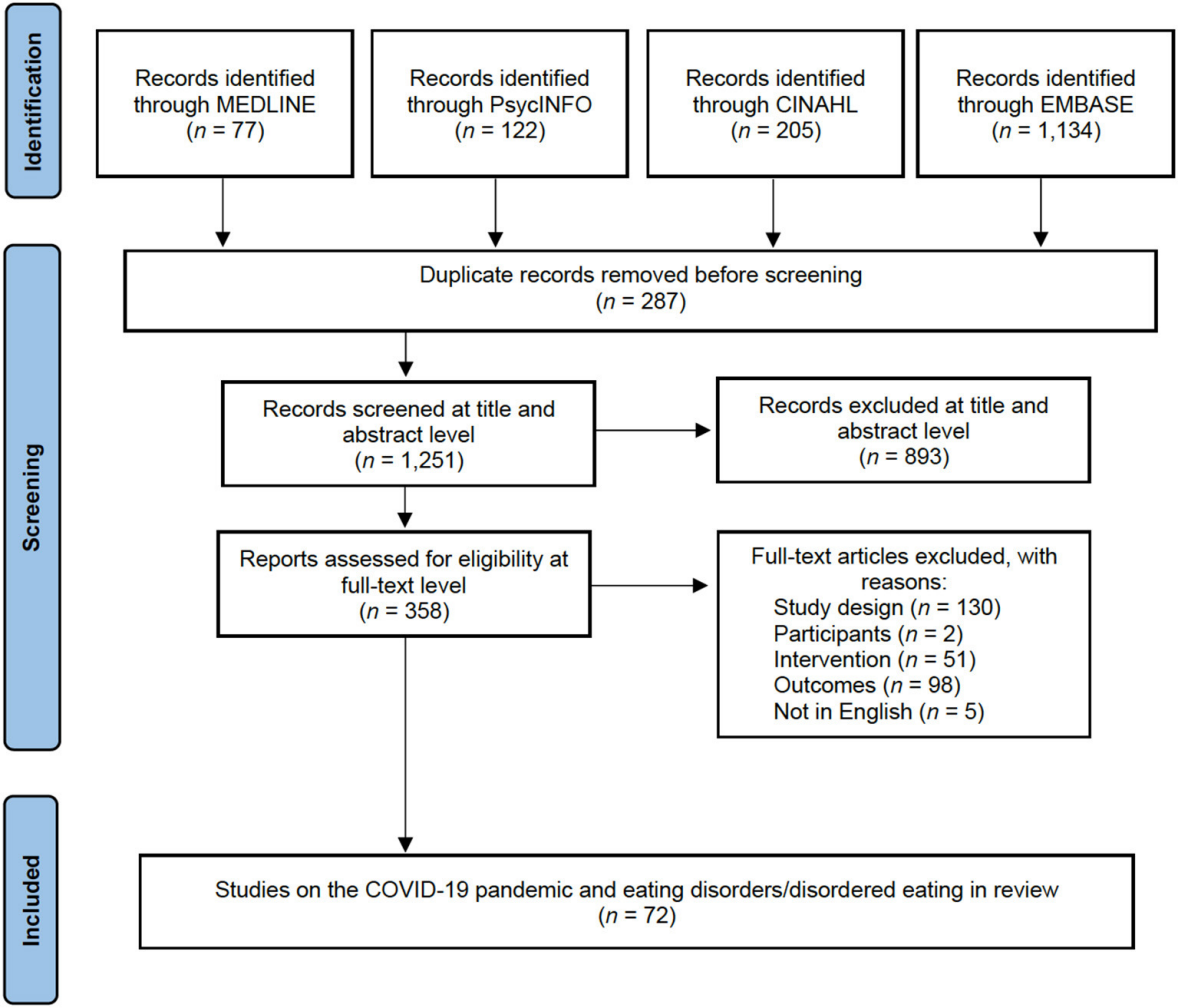
PRISMA flow diagram of study selection process.

A total of 1,538 potentially relevant studies were identified in the initial literature search. Most articles were identified through EMBASE, reflecting the large number of indexed journals held by EMBASE compared to the other searched databases. Once duplicates were removed (*n* = 287), 1,251 studies were screened at title and abstract level resulting in 893 studies deemed not relevant. The remaining 358 studies were screened for eligibility at a full-text level with 286 excluded for not meeting selection criteria. A discrepancy of one paper was identified during review author's extraction check and were resolved through consensus and subsequently included. A resulting 72 studies were included in the mixed systematic review. Three common reasons for exclusion were incorrect study design such as conference abstract, letter to the editor, and editorials, outcomes examining general eating behaviors, and studies with an incorrect intervention, such as measuring a diagnosis of COVID-19. Figure 1 presents a PRISMA flow diagram summarizing the successive steps of our selection process.

### Quality of included studies

Methodological quality of most studies (31 of 72, 43.1%) was assessed as “low” (see Supplementary material). The quality of 23 (31.9%) studies was rated as “medium” and a further 18 (25.0%) studies was considered to be “high” quality. All studies of qualitative design were rated as “high”. The most common caveats across studies were no description of response rate between respondents and non-respondents and no description of controlling for important confounding variables (e.g., age, Body Mass Index (BMI); Supplementary Table 1).

### Study characteristics

Key study characteristics of the 72 included studies are presented in Supplementary Table 1. The studies were conducted in 19 countries with most studies published in the United States (*n* = 18), Italy (*n* = 10), and the United Kingdom (*n* = 8). Study design was primarily cross-sectional, longitudinal, followed by retrospective analysis (*n* = 8). Participants predominantly comprised individuals with an eating disorder (*n* = 25), and non-clinical sample including university students (*n* = 13), and community samples (*n* = 12). Sample size of studies ranged from 10 participants in the smallest study to 12,186 participants in the largest. Sample size was highly dependent on study design (e.g., smaller sample sizes in qualitative interviews). Many studies recruited high numbers of female participants with most studies reporting a female distribution >60.0%. Mean age ranged from 9.1 (*SD* = 1.4) to 57.0 (*SD* = 14.2) years old. Ethnicity/race was reported in 22 studies, consisting of predominantly White participants in 17 studies (>60.0%). The most common tool measuring eating disorder symptoms was the Eating Disorder Examination-Questionnaire (EDE-Q; *n* = 15), Diagnostic and Statistical Manual of Mental Disorders (DSM; *n* = 9), and the Eating Attitudes Test (EAT; *n* = 6) and Eating Disorder Inventory (EDI; *n* = 6). Note that studies of mixed methods design may be reported in both quantitative and qualitative results.

### Quantitative studies

The majority of studies reported an increase of eating disorder or disordered eating behaviors during the COVID-19 pandemic. A further 31 studies reported results supporting no differences in eating disorders or disordered eating behaviors during the COVID-19 pandemic, and three studies reported a decrease in eating disorder or disordered eating behaviors (Beghi et al., 2021; Gholmie, 2021).

#### Eating disorders

There was a total of 19 quantitative studies that recruited participants with a current or previous eating disorder. Most studies reported results supporting an increase in eating disorder symptoms associated with the COVID-19 pandemic. Of note are retrospective, cross-sectional, and longitudinal findings as described below.

##### Retrospective studies

In further examination of retrospective studies, there was a reported general decline in eating disorder patient presentations at the onset of the pandemic, however this increased steadily as the pandemic progressed. Every study of retrospective design that recruited eating disorder patients reported an increase in eating disorder symptoms or presentations during the COVID-19 pandemic (Chadi et al., 2021; Matthews et al., 2021; Monteleone et al., 2021a,b; Otto et al., 2021; Springall et al., 2021). For example, Chadi et al. (2021) reported a 62% increase in annual emergency department visits for eating disorders in 2020 compared to the 2 years prior. Matthews et al. (2021) found eating disorder patients were 8.78 times more likely to be readmitted to hospital within 30 days post lockdown than pre-lockdown. Reports of 33.3–40.4% of patients suggest the onset or exacerbation of their eating disorder symptoms coincided with the COVID-19 pandemic or lockdown (Matthews et al., 2021; Springall et al., 2021).

##### Cross-sectional studies

Studies incorporating a cross-sectional design reported both a significant worsening of eating disorder symptoms during the COVID-19 pandemic (e.g., McCombie et al., 2020; Termorshuizen et al., 2020; Favreau et al., 2021), while others found no difference in symptoms during this time (e.g., Baenas et al., 2020; Branley-Bell and Talbot, 2021; Leenaerts et al., 2021; Raykos et al., 2021). We note large heterogeneity in data collection methods used across studies such as standardized eating disorder measures to researcher-created measurement scales assessing COVID-19 experiences and symptom development.

##### Longitudinal studies

Four longitudinal studies examined changes in eating disorder presentation over the COVID-19 pandemic with mixed results. For example, Ayton et al. (2022) and Beghi et al. (2021) reported an increase in admissions and referrals during the COVID-19 pandemic compared to 2019 times. Stewart et al. (2021) found no change in binge eating or major restrictions in diet in adolescents accessing mental health services during the pandemic compared to pre-pandemic. The longitudinal studies included within this mixed studies systematic review primarily met classification 4 of “level of evidence”.

#### Disordered eating behaviors

A total of 46 quantitative studies recruited participants from community samples such as university students, adolescents, school administrators, caregivers, and bariatric surgery patients. Most studies reported results supporting an increase in disordered eating behaviors associated with the COVID-19 pandemic, followed by a number of studies that reported results supporting no differences in disordered eating behaviors. Of note are cross-sectional and longitudinal findings as described below.

##### Cross-sectional studies

Among cross-sectional studies, most reported an increase in disordered eating behaviors associated with the COVID-19 pandemic. However, such results seemingly appeared to differ depending on the subscale or time period measured. For example, Elmacioglu et al. (2021) and Ramalho et al. (2021) found an increase in uncontrolled eating but not cognitive restraint associated with the COVID-19 pandemic. Flaudias et al. (2020) found high lockdown-related stress was associated with binge eating and dietary restraint over the past week, but not related with COVID-19 in general. There were a range of disordered eating behavior outcomes measured such as binge eating (e.g., Albert et al., 2021; Athanasiadis et al., 2021), weight and shape concerns (e.g., Coimbra et al., 2021), uncontrolled eating (e.g., Elmacioglu et al., 2021; Ramalho et al., 2021), and compensatory exercise behaviors (e.g., Phillipou et al., 2021).

##### Longitudinal studies

Six studies longitudinally assessed disordered eating behaviors over the COVID-19 pandemic in community samples. Most studies reported an increase in disordered eating behaviors, including binge eating, weight concern, and shape concern, associated with the COVID-19 pandemic compared to pre-pandemic data. On the other hand, Conceição et al. (2021) found significantly higher rates of weight concern, but not shape concern, restriction, or food concerns in bariatric surgery patients at 3-year surgery follow-up compared to controls. Martínez-de-Quel et al. (2021) reported no significant differences in eating disorder risk before or during COVID-19 lockdown.

### Qualitative studies

In studies that incorporated qualitative methods, such as interviews and content analysis, the majority of studies reported an exacerbation of eating disorders symptoms or increased disordered eating behaviors (Clark Bryan et al., 2020; McCombie et al., 2020; Richardson et al., 2020; Branley-Bell and Talbot, 2021; Brown et al., 2021; Brownstone et al., 2021; Frayn et al., 2021; Giel et al., 2021; Hunter and Gibson, 2021; Nutley et al., 2021; Phillipou et al., 2021; Simone et al., 2021; Zeiler et al., 2021). Four studies addressed the positive aspects of the COVID-19 pandemic which allowed participants to reflect and work on their eating disorder symptoms resulting in an improvement of their illness (McCombie et al., 2020; Frayn et al., 2021; Hunter and Gibson, 2021; Zeiler et al., 2021). Two studies reported no differences in eating disorder or disordered eating behaviors associated with the COVID-19 pandemic (Muzi et al., 2021; Raykos et al., 2021). A description of the main themes and subthemes of included qualitative studies is provided in Supplementary material.

#### Eating disorders

Ten out of 15 qualitative interview studies recruited individuals with an eating disorder or lived eating disorder experience (Clark Bryan et al., 2020; McCombie et al., 2020; Branley-Bell and Talbot, 2021; Brown et al., 2021; Frayn et al., 2021; Giel et al., 2021; Hunter and Gibson, 2021; Zeiler et al., 2021). Most studies reported an increased in eating disorder symptoms or contacts associated with the COVID-19 pandemic. Main themes associated with the COVID-19 pandemic included loss of control of eating, restrictions in accessing professional support, symptom improvement and deterioration, attempts to self-manage recovery, increased general stress and anxiety, and positive aspects to life in lockdown such as less pressure to take part in social events. One study found no differences in the reduction of disordered eating symptoms in eating disorder patients during the pandemic (Raykos et al., 2021).

#### Disordered eating behaviors

A total of five studies using qualitative design (i.e., interviews and content analysis) assessed disordered eating behaviors in community samples such as online reddit (social media platform) users and adolescents/young adults. Most studies reported an increase in disordered eating behaviors associated with the COVID-19 pandemic (Brownstone et al., 2021; Nutley et al., 2021; Phillipou et al., 2021; Simone et al., 2021). Reported disordered eating behaviors include binge eating, body dissatisfaction, exercise behaviors, “mindless” eating, and eating to “cope”. Two studies reported no differences in disordered eating behaviors (Muzi et al., 2021; Phillipou et al., 2021), for example Phillipou et al. (2021) found binge eating, purging, and exercise behaviors did not differ between COVID-19 waves.

### Studies with understudied or minority groups

#### Children and adolescents

Ten studies examined the impact of the COVID-19 pandemic on eating disorders and disordered eating behaviors in adolescents and/or young people (Chadi et al., 2021; Koenig et al., 2021; Lin et al., 2021; Matthews et al., 2021; Muzi et al., 2021; Otto et al., 2021; Springall et al., 2021; Stewart et al., 2021; Wang L. et al., 2021; Zeiler et al., 2021). Five studies used retrospective analysis or chart review to examine eating disorder presentations in hospital or inpatient facilities finding the COVID-19 pandemic was associated with increased visits from young people requiring acute medical intervention (Chadi et al., 2021; Lin et al., 2021; Matthews et al., 2021; Otto et al., 2021; Springall et al., 2021). Four studies assessed disordered eating behaviors with the majority finding no significant differences in disordered eating scores pre- and post-pandemic. Lastly, one study was qualitative in design, highlighting main themes associated with the COVID-19 pandemic in adolescent patients with anorexia nervosa (Zeiler et al., 2021). The study highlighted both deteriorations and improvements in patients eating disorder symptoms, for example an increase in anorexia nervosa-related cognitions and behaviors exacerbated by feeling isolated at home and observed by family members, but also less stress due to attending school and socializing provided patients time for greater consideration to their “personal needs”.

#### Across the gender spectrum

One study by Brownstone et al. (2021) in the United States examined the impact of the COVID-19 pandemic on eating disorders and disorder eating behaviors in transgender and gender non-binary individuals using a qualitative approach. The authors found the majority of participants reported an increase in disordered eating behaviors which related to struggles in losing gender affirming spaces, work and financial security, increased time along exacerbating gender dysphoria, and increased exhaustion in advocating for social justice in holding a minority identity. Participants also reported benefits from the pandemic in improving their disordered eating tied to finding affirming online spaces and accessing different pathways to recovery.

## Discussion

This mixed studies systematic review expands on previous reviews (e.g., Sideli et al., 2021; Linardon et al., 2022) to examine the impact of the COVID-19 pandemic on eating disorders and disordered eating behaviors across the age spectrum. In doing so, we provide an insight into pathological eating behaviors in individuals with and without a diagnosed eating disorder. As vaccinations become more available and restrictions have begun to ease around the world, this mixed systematic review expands on previous reviews by providing a timely insight into past literature and help anticipate possible future impacts of the COVID-19 pandemic on eating disorders and disordered eating behaviors. We identified a total of 72 studies with most being cross-sectional in nature and published in the United States. Participants mainly comprised of individuals with an eating disorder or university samples of White women. Overall, we found a range of longitudinal, cross-sectional, and retrospective studies reported an increased trend in eating disorders and disordered eating behaviors associated with the COVID-19 pandemic. We also found a portion of studies reported no differences in eating disorder or disordered eating behaviors associated with the COVID-19 pandemic supporting reports of both positive and negative consequences of the pandemic. Ultimately, the mixed studies design of this systematic review allows us to differentiate these findings by comparing both qualitative and quantitative results.

The literature showed, mostly, that individuals with an eating disorder were likely to experience worsening of symptoms during the COVID-19 pandemic. It is possible that changes in social and home environment, coupled with self-isolation and disruptions in accessing healthcare, increased the risk of relapse or worsening of symptoms for these groups. For example, individuals may have experienced difficulties in maintaining compensatory behaviors such as compulsive physical exercise due to lockdown mandates, worsening weight and shape preoccupation, and therefore exacerbating eating disorder symptoms. Notably, a theme reported across qualitative studies was that the COVID-19 pandemic provided individuals with an eating disorder space and time for reflection. Participants described this period as an opportunity to reflect on their eating disorder recovery progress and experiences, and the challenges that remained ahead of them in their recovery. Hunter and Gibson (2021) aptly describe this time as a “mixed bag” of positive and negative experiences for individuals with an eating disorder.

The majority of the cross-sectional and longitudinal literature reviewed suggest that the COVID-19 pandemic showed a positive association with disordered eating behaviors among the general population. In particular, our findings show these disordered eating behaviors, such as binge eating, weight and shape concerns, and uncontrolled eating, may have played a large role in coping during this time. While the relationship with disordered eating shows greater variation than the results from eating disorder patient studies, it nonetheless supports the idea that changes to social and home environment may possibly be common triggers to disordered eating behaviors (Mazzeo and Bulik, 2008). The greater variation between eating disorder and disordered eating behaviors results could be explained by a heavy reliance on cross-sectional design studies and diverse samples. For example, studies assessing disordered eating often consisted of university students or other specific clinical groups such as family caregivers of people with dementia, bariatric surgery patients, and individuals with celiac disease. Furthermore, most studies reporting no differences in disordered eating behaviors used self-report data (i.e., EDE-Q) and convenience sampling.

Several studies investigated the COVID-19 pandemic in terms of its impact on stress on disordered eating behaviors. For example, Flaudias et al. (2020) reported higher stress during lockdown was associated with higher likelihood of binge eating. Czepczor-Bernat et al. (2021) and Jordan et al. (2021) found COVID-19-related stress was positively associated with increased disordered eating. While the role of stress in the development and maintenance of eating disorders has been long established (Troop et al., 1998; Lobera et al., 2009), it is thought that the COVID-19 pandemic led to an increase in intensity of stress and uncertainty, bringing about increased disordered eating as a means to cope (Flaudias et al., 2020).

This mixed systematic review provides a novel perspective aimed at assessing the impact of the COVID-19 pandemic and eating disorders and disordered eating behaviors in understudied and minority groups such as children and adolescents, geriatric populations, people from culturally and linguistically diverse backgrounds, LGBTIQA+ community, Indigenous peoples, and across the gender spectrum. A total of ten studies were found to assess the impact of the pandemic in children and adolescents, and a further one study assessed the impact in people across the gender spectrum. Specifically, it is likely that children and adolescents present a vulnerable population to the impacts of the COVID-19 pandemic on eating disorders and disordered eating behaviors (Sharp et al., 2017). Qualitative interviews with adolescents with eating disorders suggest a restriction in personal freedom in terms of home-schooling and feelings of imprisonment at home with family members appears to be a unique risk factor for this population (Zeiler et al., 2021). Thus, highly accessible online eating disorder intervention and prevention programs may be helpful for youths, particularly addressing healthy coping strategies that can be employed within the home environment (Beilharz et al., 2021).

Among transgender and non-binary individuals, the COVID-19 pandemic created additional complex experiences that exacerbated body and eating concerns in these groups (Brownstone et al., 2021). Specifically, while participants reported a loss of affirming spaces during the COVID-19 pandemic, few found new supportive spaces online, such as transgender and non-binary online eating disorder support groups (Brownstone et al., 2021). This study suggests a need for both online and in-person gender-inclusive spaces, not just for eating disorder support but also more broadly for gender identity, to provide recognition and support for these groups during the pandemic. Importantly this study highlights the complex and intersectional ways the pandemic has influenced transgender and gender non-binary individuals' experiences with disordered eating. Taken together, this single study, and the lack of additional studies focusing on minority groups, provides preliminary evidence for the degree to which minority groups may have been neglected in the eating disorder/COVID-19 pandemic research space to date, and therefore additional research is needed in these specific groups.

### Clinical implications and recommendations

Findings from this mixed systematic review suggest individuals with an eating disorder are some of the most vulnerable groups to the impacts of the COVID-19 pandemic. Increased stress and uncertainty associated with the pandemic have potentially created a “perfect storm” for eating disorder deterioration. Some people who have previously been in “recovery” have relapsed and others at risk have developed new eating disorders. Early intervention is key to reducing symptom severity and promoting recovery (Flynn et al., 2021). However, specialized eating disorder treatment services around the world are mostly overwhelmed with demand and so people are often waiting long periods for evidence-based in-person or telehealth treatment with health professionals. Online treatment programs with supported monitoring of physical health symptoms in an outpatient setting may be a suitable option for some while waiting (Beilharz et al., 2021; Moghimi et al., 2021). Specifically, the COVID-19 pandemic has allowed for the proliferation of well-researched digital and e-health mental health tools, which have shown to be highly feasible and acceptable in eating disorder populations. We call for additional investments to support the building of such alternative eating disorder treatment interventions (e.g., online guided self-help programs) to relieve unprecedented workload on health professionals (Fernando and Sharp, 2020; Allison et al., 2021).

Our findings also suggest that the COVID-19 pandemic created additional demands for carers of people with an eating disorder. For example, organizing and managing a new daily routine and creating “distractions” for their loved one. Our findings also imply these demands extended to concerns regarding the progression of their treatment during the lockdown period due to reduced outpatient monitoring and increased use of telehealth (Clark Bryan et al., 2020; Zeiler et al., 2021). In some carer's experiences, these fears may have been exacerbated by feeling “left out” or uninformed by the treating team. Ongoing carer support may be useful in validating the additional challenges and demands experienced by carers due to the COVID-19 pandemic.

Given the results of our mixed systematic review suggest an increase in disordered eating in the community associated with the pandemic, it is important that primary care workers and other professionals in settings that support early identification and intervention (e.g., teachers, sport coaches) remain vigilant of these behaviors, particularly as they are often the first point of contact. While even symptomatic eating disorder behaviors can often be misdiagnosed, primary healthcare providers should be attentive to disordered eating behaviors such as weight and eating concerns, but also to the risks associated with disordered eating such as gastrointestinal problems, bone loss, and increased anxiety and depression. In doing so, primary healthcare providers will be able to detect these early warning signs and provide appropriate referral for early intervention and care. Furthermore, given the findings related to the impact of the pandemic on carers of those with eating disorders, it is recommended that primary healthcare professionals screen for increased stress, and potentially mood and anxiety disorders amongst carers. Finally, the emergence of the COVID-19 pandemic has placed additional challenges on mental health clinicians. We encourage clinicians to be creative in how they manage these challenges such as arranging weight reviews through a loved one. Ultimately, increased challenges and workloads on clinicians have increased risk of burnout (Greenberg et al., 2020), and so clinicians and services should be mindful of this situation.

### Limitations and future research

There are several limitations to this mixed systematic review. First, most studies were of overall low quality, indicating a high degree of risk of bias. Specifically, most cross-sectional studies were of low quality, in comparison to all qualitative studies rated as high quality, which is likely indicative of the brisk rate at which COVID-19 related literature were being published leading to methodological quality concerns in the field (Jung et al., 2021). As such, the findings of this mixed systematic review must be considered in light of the limited high-quality evidence. Further to this, we encourage the use of pre-registration of study protocols and open data to reduce the overabundance of studies reporting on similar results. Most studies were also cross-sectional meaning the casual relationship between the COVID-19 pandemic and eating disorders and disordered eating behaviors cannot be established in these studies. Future research should pay particular attention to longitudinal or case-control studies to examine the future impacts of the COVID-19 pandemic, while also controlling for relevant socio-demographic variables which was a common caveat when assessing methodological quality of studies.

Another limitation is the limited variability among demographic variables with participants primarily comprising young to middle-aged White women. While eating disorders and disordered eating behaviors are disproportionately more common in women than men (Striegel-Moore et al., 2009), it remains important to examine their effects in a wide range of demographics including gender identities, sexual orientation, ethnicities, and socio-economic status. Furthermore, there were no studies assessing the impact of the COVID-19 pandemic on eating disorders and disordered eating behaviors in geriatric populations, culturally and linguistically diverse backgrounds, LGBTIQA+ communities, and indigenous populations. It is clear that broad representation of understudied and minority groups continues to be systematic limitation across the eating disorder and disordered eating research field, and therefore remains a crucial area of future research. In developing our understanding of understudied and minority groups as they relate to eating disorders, we can begin tackling barriers to the identification and treatment of eating disorders in these groups. Finally, future high-quality research is also needed to examine the long-term implications of the COVID-19 pandemic on eating disorders and disordered eating behaviors (Tan et al., 2020). It is possible that the COVID-19 pandemic will have a long-term mental toll on individuals who had particularly distressing experiences during this time.

## Conclusion

This mixed systematic review expands on previous literature (e.g., Linardon et al., 2022) to demonstrate the COVID-19 pandemic has likely led to an increase in eating disorders and disordered eating behaviors. Specifically, this mixed systematic review highlights potentially vulnerable groups, such as children and adolescents and those with a history of an eating disorder, to the impacts of the COVID-19 pandemic. In light of the overall low-quality studies considered in this review, high-quality research is vital in understanding the long-term impacts of the COVID-19 pandemic on eating disorders and disordered eating behaviors.

## Data availability statement

The original contributions presented in the study are included in the article/Supplementary material, further inquiries can be directed to the corresponding author/s.

## Author contributions

CM and GS designed the study. CM performed the systematic search, data extraction, and initial draft of the manuscript. GS conducted extraction and quality bias checks. CM, RU, and GS conducted drafting of the manuscript revisions and the final version of the manuscript. All authors contributed to the article and approved the submitted version.

## Funding

Funding was received by the Butterfly Foundation to undertake this systematic review.

## Conflict of interest

Author RU was employed by the Butterfly Foundation. The remaining authors declare that the research was conducted in the absence of any commercial or financial relationships that could be construed as a potential conflict of interest.

## Publisher's note

All claims expressed in this article are solely those of the authors and do not necessarily represent those of their affiliated organizations, or those of the publisher, the editors and the reviewers. Any product that may be evaluated in this article, or claim that may be made by its manufacturer, is not guaranteed or endorsed by the publisher.

## References

[B1] Albert U. Losurdo P. Leschiutta A. Macchi S. Samardzic N. Casaganda B. . (2021). Effect of SARS-CoV-2 (COVID-19) pandemic and lockdown on body weight, maladaptive eating habits, anxiety, and depression in a bariatric surgery waiting list cohort. Obes. Surg. 31, 1905–1911. 10.1007/s11695-021-05257-5 33611765 PMC7896875

[B2] Allison S. Wade T. Schmidt U. Treasure J. Bastiampillai T. Looi J. C. L. (2021). Setting a youth-focused research agenda for eating disorders during the COVID-19 pandemic. Aust New Zeal J Psychiatry. 56, 591–593. 10.1177/00048674211054743 34702094

[B3] Arcelus J. Mitchell A. J. Wales J. Nielsen S. (2011). Mortality rates in patients with anorexia nervosa and other eating disorders. A meta-analysis of 36 studies. Arch Gen Psychiatry 68, 724–731. 10.1001/archgenpsychiatry.2011.74 21727255

[B4] Athanasiadis D. I. Hernandez E. Hilgendorf W. Roper A. Embry M. Selzer D. . (2021). How are bariatric patients coping during the coronavirus disease 2019 (COVID-19) pandemic Analysis of factors known to cause weight regain among postoperative bariatric patients. Surg. Obes. Relat. Dis. 17, 756–764. 10.1016/j.soard.2020.11.021 33390351 PMC7699156

[B5] Ayton A. Viljoen D. Ryan S. Ibrahim A. Ford D. (2022). Risk, demand, capacity and outcomes in adult specialist eating disorder services in South-East of England before and since COVID-19. BJPsych Bull. 46, 89–95. 10.1192/bjb.2021.73 34486966 PMC9074142

[B6] Baenas I. Caravaca-Sanz E. Granero R. Sánchez I. Riesco N. Testa G. . (2020). COVID-19 and eating disorders during confinement: analysis of factors associated with resilience and aggravation of symptoms. Eur. Eat. Disord. Rev. 28, 855–863. 10.1002/erv.2771 32815293 PMC7461472

[B7] Beat (2021). Helpline Demand Soars With 28% of New Contacts Noting Coronavirus as a Trigger. Retrieved from: https://www.beateatingdisorders.org.uk/news/beat-news/helpline-demand-soars/ (accessed November 23, 2021).

[B8] Beghi M. Brandolini R. Casolaro I. Beghi E. Cornaggia C. M. Fraticelli C. . (2021). Effects of lockdown on emergency room admissions for psychiatric evaluation: an observational study from the AUSL Romagna, Italy. Int. J. Psychiatry Clin. Pract. 25, 135–139. 10.1080/13651501.2020.1859120 33346685

[B9] Beilharz F. Sukunesan S. Rossell S. L. Kulkarni J. Sharp G. (2021). Development of a positive body image chatbot (KIT) with young people and parents/carers: qualitative focus group study. J. Med. Internet Res. 23, e27807. 10.2196/27807 34132644 PMC8277317

[B10] Branley-Bell D. Talbot C. V. (2021). “It is the only constant in what feels like a completely upside down and scary world”: living with an eating disorder during COVID-19 and the importance of perceived control for recovery and relapse. Appetite 167, 105596. 10.1016/j.appet.2021.105596 34252493 PMC8423590

[B11] Brown S. Opitz M.-C. Peebles A. I. Sharpe H. Duffy F. Newman E. (2021). A qualitative exploration of the impact of COVID-19 on individuals with eating disorders in the UK. Appetite 156, 104977. 10.1016/j.appet.2020.104977 32991945 PMC7521890

[B12] Brownstone L. M. Kelly D. A. Maloul E. K. Dinneen J. L. Palazzolo L. A. Raque T. L. . (2021). “It's just not comfortable to exist in a body”: transgender/gender nonbinary individuals' experiences of body and eating distress during the COVID-19 pandemic. Psychol. Sex. Orient. Gender Divers. 10.1037/sgd0000519

[B13] Butterfly Foundation (2020). Body Image Concerns and Eating Disorders Exacerbated by COVID-19. Retrieved from: https://butterfly.org.au/news/body-image-concerns-eating-disorders-exacerbated-by-covid-19/ (accessed October 4, 2021).

[B14] Chadi N. Piano C. S.-D. Osmanlliu E. Gravel J. Drouin O. (2021). Mental health-related emergency department visits in adolescents before and during the COVID-19 pandemic: a multicentric retrospective study. J. Adolesc. Health. 69, 847–850. 10.1016/j.jadohealth.2021.07.036 34462192 PMC8421028

[B15] Clark Bryan D. Macdonald P. Ambwani S. Cardi V. Rowlands K. Willmott D. . (2020). Exploring the ways in which COVID-19 and lockdown has affected the lives of adult patients with anorexia nervosa and their carers. Eur. Eat. Disord. Rev. 28, 826–835. 10.1002/erv.2762 32643844 PMC7362064

[B16] Coimbra M. Paixão C. Ferreira C. (2021). Exploring eating and exercise-related indicators during COVID-19 quarantine in Portugal: concerns and routine changes in women with different BMI. Eat. Weight Disord. 27, 225–232. 10.21203/rs.3.rs-114924/v1 33751463 PMC7982514

[B17] Conceição E. de Lourdes M. Ramalho S. Félix S. Pinto-Bastos A. Vaz A. R. (2021). Eating behaviors and weight outcomes in bariatric surgery patients amidst COVID-19. Surg. Obes. Relat. Dis. 17, 1165–1174. 10.1016/j.soard.2021.02.025 33812789 PMC7908843

[B18] Czepczor-Bernat K. Swami V. Modrzejewska A. Modrzejewska J. (2021). COVID-19-related stress and anxiety, body mass index, eating disorder symptomatology, and body image in women from Poland: a cluster analysis approach. Nutrients 13, 1384. 10.3390/nu13041384 33924010 PMC8073902

[B19] Dumitracu M. andru F. Carsote M. Petca R. Gheorghisan-galateanu A. Petca A. . (2021). Anorexia nervosa: COVID-19 pandemic period (Review). Exp. Ther. Med. 22, 804–804. 10.3892/etm.2021.10236 34093760 PMC8170656

[B20] Elmacioglu F. Emiroglu E. Ülker M. T. Özyilmaz Kircali B. Oruç S. (2021). Evaluation of nutritional behaviour related to COVID-19. Public Health Nutr. 24, 512–518. 10.1017/S1368980020004140 33070798 PMC7737137

[B21] Esterhuizen T. M. Thabane L. (2016). Con: meta-analysis: some key limitations and potential solutions. Nephrol. Dialysis Transplant. 31, 882–885. 10.1093/ndt/gfw092 27217394

[B22] Favreau M. Hillert A. Osen B. Gärtner T. Hunatschek S. Riese M. . (2021). Psychological consequences and differential impact of the COVID-19 pandemic in patients with mental disorders. Psychiatry Res. 302, 114045. 10.1016/j.psychres.2021.114045 34126461 PMC8180351

[B23] Fernando A. N. Sharp G. (2020). Genital self-image in adolescent girls: the effectiveness of a brief educational video. Body Image 35, 75–83. 10.1016/j.bodyim.2020.08.007 33011539

[B24] Flaudias V. Iceta S. Zerhouni O. Rodgers R. F. Billieux J. Llorca P.-M. . (2020). COVID-19 pandemic lockdown and problematic eating behaviors in a student population. J. Behav. Addict. 9, 826–835. 10.1556/2006.2020.00053 32976112 PMC8943668

[B25] Flynn M. Austin A. Lang K. Allen K. Bassi R. Brady G. . (2021). Assessing the impact of first episode rapid early intervention for eating disorders on duration of untreated eating disorder: a multi-centre quasi-experimental study. Eur. Eat. Disord. Rev. 29, 458–471. 10.1002/erv.2797 33112472

[B26] Frayn M. Fojtu C. Juarascio A. (2021). COVID-19 and binge eating: patient perceptions of eating disorder symptoms, tele-therapy, and treatment implications. Curr. Psychol. 40, 6249–6258. 10.1007/s12144-021-01494-0 33623352 PMC7891466

[B27] Gholmie Y. (2021). Disordered Eating Attitudes and Behaviors in Individuals With Celiac Disease and the Association With Quality of Life. ProQuest Dissertations Publishing. New York, NY: Colombia Academic Commons.

[B28] Giel K. E. Schurr M. Zipfel S. Junne F. Schag K. (2021). Eating behaviour and symptom trajectories in patients with a history of binge eating disorder during COVID-19 pandemic. Eur. Eat. Disord. Rev. 29, 657–662. 10.1002/erv.2837 33955610 PMC8206923

[B29] Gordon C. M. Katzman D. K. (2020). Lessons learned in caring for adolescents with eating disorders: the Singapore experience. J. Adolesc. Health 67, 5–6. 10.1016/j.jadohealth.2020.03.041 32376159 PMC7141451

[B30] Greenberg N. Docherty M. Gnanapragasam S. Wessely S. (2020). Managing mental health challenges faced by healthcare workers during covid-19 pandemic. BMJ 368, m1211. 10.1136/bmj.m1211 32217624

[B31] Hong Q. N. Fàbregues S. Bartlett G. Boardman F. Cargo M. Dagenais P. . (2018). The mixed methods appraisal tool (MMAT) version 2018 for information professionals and researchers. Educ. Inf. 34, 285–291. 10.3233/EFI-180221

[B32] Hunter R. Gibson C. (2021). Narratives from within ‘lockdown': a qualitative exploration of the impact of COVID-19 confinement on individuals with anorexia nervosa. Appetite 166, 105451. 10.1016/j.appet.2021.105451 34171411 PMC9756092

[B33] Jarvis C. I. Van Zandvoort K. Gimma A. Prem K. Klepac P. Rubin G. J. . (2020). Quantifying the impact of physical distance measures on the transmission of COVID-19 in the UK. BMC Med. 18, 124. 10.1186/s12916-020-01597-8 32375776 PMC7202922

[B34] Jordan A. K. Barnhart W. R. Studer-Perez E. I. Kalantzis M. A. Hamilton L. Musher-Eizenman D. R. (2021). ‘Quarantine 15': pre-registered findings on stress and concern about weight gain before/during COVID-19 in relation to caregivers' eating pathology. Appetite 166, 105580. 10.1016/j.appet.2021.105580 34186158 PMC9756091

[B35] Jung R. G. Di Santo P. Clifford C. Prosperi-Porta G. Skanes S. Hung A. . (2021). Methodological quality of COVID-19 clinical research. Nat. Commun. 12, 943. 10.1038/s41467-021-21220-5 33574258 PMC7878793

[B36] Koenig J. Kohls E. Moessner M. Lustig S. Bauer S. Becker K. . (2021). The impact of COVID-19 related lockdown measures on self-reported psychopathology and health-related quality of life in German adolescents. Eur. Child Adolesc. Psychiatry. 10.1007/s00787-021-01843-1 34247297 PMC8272610

[B37] Kumar A. Nayar K. R. (2021). COVID 19 and its mental health consequences. J. Mental Health 30, 1–2. 10.1080/09638237.2020.1757052 32339041

[B38] Leenaerts N. Vaessen T. Ceccarini J. Vrieze E. (2021). How COVID-19 lockdown measures could impact patients with bulimia nervosa: exploratory results from an ongoing experience sampling method study. Eat. Behav. 41, 101505. 10.1016/j.eatbeh.2021.101505 33831813 PMC9759935

[B39] Li Y. Scherer N. Felix L. Kuper H. (2021). Prevalence of depression, anxiety and post-traumatic stress disorder in health care workers during the COVID-19 pandemic: a systematic review and meta-analysis. PLoS ONE 16, e0246454. 10.1371/journal.pone.0246454 33690641 PMC7946321

[B40] Liberati A. Altman D. G. Tetzlaff J. Mulrow C. Gøtzsche P. C. Ioannidis J. P. A. . (2009). The PRISMA statement for reporting systematic reviews and meta-analyses of studies that evaluate healthcare interventions: explanation and elaboration. BMJ 339, b2700. 10.1136/bmj.b2700 19622552 PMC2714672

[B41] Lin J. A. Hartman-Munick S. M. Kells M. R. Milliren C. E. Slater W. A. Woods E. R. . (2021). The impact of the COVID-19 pandemic on the number of adolescents/young adults seeking eating disorder-related care. J. Adolesc. Health 69, 660–663. 10.1016/j.jadohealth.2021.05.019 34266715 PMC8415773

[B42] Linardon J. Messer M. Rodgers R. F. Fuller-Tyszkiewicz M. (2022). A systematic scoping review of research on COVID-19 impacts on eating disorders: a critical appraisal of the evidence and recommendations for the field. Int. J. Eat. Disord. 55, 3–38. 10.1002/eat.23640 34773665 PMC8646470

[B43] Lobera I. J. Estébanez S. Fernández M. J. S. Bautista E. Á. Garrido O. (2009). Coping strategies in eating disorders. Eur. Eat. Disord. Rev. 17, 220–226. 10.1002/erv.920 19274619

[B44] Martínez-de-Quel Ó. Suárez-Iglesias D. López-Flores M. Pérez C. A. (2021). Physical activity, dietary habits and sleep quality before and during COVID-19 lockdown: a longitudinal study. Appetite 158, 105019. 10.1016/j.appet.2020.105019 33161046 PMC8580211

[B45] Matthews A. Kramer R. A. Peterson C. M. Mitan L. (2021). Higher admission and rapid readmission rates among medically hospitalized youth with anorexia nervosa/atypical anorexia nervosa during COVID-19. Eat. Behav. 43, 101573. 10.1016/j.eatbeh.2021.101573 34619464 PMC8490008

[B46] Mazzeo S. E. P. Bulik C. M. P. (2008). Environmental and genetic risk factors for eating disorders: what the clinician needs to know. Child Adolesc. Psychiatr. Clin. N. Am. 18, 67–82. 10.1016/j.chc.2008.07.003 19014858 PMC2719561

[B47] McCombie C. Austin A. Dalton B. Lawrence V. Schmidt U. (2020). “Now It's Just Old Habits and Misery”-understanding the impact of the Covid-19 pandemic on people with current or life-time eating disorders: a qualitative study. Front. Psychiatry 11, 589225. 10.3389/fpsyt.2020.589225 33192736 PMC7653176

[B48] Miniati M. Marzetti F. Palagini L. Marazziti D. Orru G. Conversano C. . (2021). Eating disorders spectrum during COVID pandemic: a systematic review. Front. Psychol. 12, 663376. 10.3389/fpsyg.2021.663376 34658992 PMC8511307

[B49] Moghimi E. Davis C. Rotondi M. (2021). the Efficacy of eHealth interventions for the treatment of adults diagnosed with full or subthreshold binge eating disorder: systematic review and meta-analysis. J. Med. Int. Res. 23, e17874. 10.2196/17874 34283028 PMC8335602

[B50] Moher D. Liberati A. Tetzlaff J. Altman D. G. (2009). Preferred reporting items for systematic reviews and meta-analyses: the PRISMA statement. BMJ 339, 332–336. 10.1136/bmj.b2535 21603045 PMC3090117

[B51] Monteleone A. M. Cascino G. Marciello F. Abbate-Daga G. Baiano M. Balestrieri M. . (2021a). Risk and resilience factors for specific and general psychopathology worsening in people with Eating Disorders during COVID-19 pandemic: a retrospective Italian multicentre study. Eat. Weight Disord. 26, 2443–2452. 10.1007/s40519-020-01097-x 33426630 PMC7797193

[B52] Monteleone A. M. Marciello F. Cascino G. Abbate-Daga G. Anselmetti S. Baiano M. . (2021b). The impact of COVID-19 lockdown and of the following “re-opening” period on specific and general psychopathology in people with Eating Disorders: the emergent role of internalizing symptoms. J. Affect. Disord. 285, 77–83. 10.1016/j.jad.2021.02.037 33636674 PMC9755808

[B53] Muzi S. Sansò A. Pace C. S. (2021). What's happened to Italian adolescents during the COVID-19 pandemic A preliminary study on symptoms, problematic social media usage, and attachment: relationships and differences with pre-pandemic peers. Front. Psychiatry 12, 590543. 10.3389/fpsyt.2021.590543 33986698 PMC8110826

[B54] Nearchou F. Flinn C. Niland R. Subramaniam S. S. Hennessy E. (2020). Exploring the impact of COVID-19 on mental health outcomes in children and adolescents: a systematic review. Int. J. Environ. Res. Public Health 17, 8479. 10.3390/ijerph17228479 33207689 PMC7698263

[B55] Necho M. Tsehay M. Birkie M. Biset G. Tadesse E. (2021). Prevalence of anxiety, depression, and psychological distress among the general population during the COVID-19 pandemic: a systematic review and meta-analysis. Int. J. Soc. Psychiatry. 7, 892–906. 10.1177/00207640211003121 33794717

[B56] Nutley S. K. Falise A. M. Henderson R. Apostolou V. Mathews C. A. Striley C. W. (2021). Impact of the COVID-19 pandemic on disordered eating behavior: qualitative analysis of social media posts. JMIR Mental Health 8, e26011. 10.2196/26011 33465035 PMC7842857

[B57] Otto A. K. Jary J. M. Sturza J. Miller C. A. Prohaska N. Bravender T. . (2021). Medical admissions among adolescents with eating disorders during the COVID-19 pandemic. Pediatrics 148:e2021052201. 10.1542/peds.2021-052201 34244452

[B58] Pereira R. F. Alvarenga M. (2007). Disordered eating: identifying, treating, preventing, and differentiating it from eating disorders. Diabetes Spectr. 20, 141–148. 10.2337/diaspect.20.3.141

[B59] Petticrew M. Egan M. Thomson H. Hamilton V. Kunkler R. Roberts H. (2008). Publication bias in qualitative research: what becomes of qualitative research presented at conferences J. Epidemiol. Community Health 62, 552–551. 10.1136/jech.2006.059394 18477755

[B60] Phillipou A. Meyer D. Neill E. Tan E. J. Toh W. L. Van Rheenen T. E. . (2020). Eating and exercise behaviors in eating disorders and the general population during the COVID-19 pandemic in Australia: initial results from the COLLATE project. Int. J. Eat. Disord. 53, 1158–1165. 10.1002/eat.23317 32476163 PMC7300745

[B61] Phillipou A. Tan E. J. Toh W. L. Van Rheenen T. E. Meyer D. Neill E. . (2021). Mental health of individuals with and without eating disorders across six months and two waves of COVID-19. Eat. Behav. 43, 101564. 10.1016/j.eatbeh.2021.101564 34509935 PMC8443406

[B62] Ramalho S. M. Trovisqueira A. de Lourdes M. Gonçalves S. Ribeiro I. Vaz A. R. . (2021). The impact of COVID-19 lockdown on disordered eating behaviors: the mediation role of psychological distress. Eat. Weight Disord. 10.21203/rs.3.rs-98867/v2 33713336 PMC7955211

[B63] Raykos B. C. Erceg-Hurn D. M. Hill J. Campbell B. N. C. McEvoy P. M. (2021). Positive outcomes from integrating telehealth into routine clinical practice for eating disorders during COVID-19. Int. J. Eat. Disord. 54, 1689–1695. 10.1002/eat.23574 34184797

[B64] Reba-Harrelson L. Von Holle A. Hamer R. M. Swann R. Reyes M. L. Bulik C. M. (2009). Patterns and prevalence of disordered eating and weight control behaviors in women ages 25–45. Eat. Weight Disord. 14, e190–e198. 10.1007/BF03325116 20179405 PMC3612547

[B65] Reinders Folmer C. P. Brownlee M. A. Fine A. D. Kooistra E. B. Kuiper M. E. Olthuis E. H. . (2021). Social distancing in America: understanding long-term adherence to COVID-19 mitigation recommendations. PLoS ONE 16, e0257945. 10.1371/journal.pone.0257945 34559863 PMC8462713

[B66] Richardson C. Patton M. Phillips S. Paslakis G. (2020). The impact of the COVID-19 pandemic on help-seeking behaviors in individuals suffering from eating disorders and their caregivers. Gen. Hosp. Psychiatry 67, 136–140. 10.1016/j.genhosppsych.2020.10.006 33129138 PMC10277602

[B67] Sharp G. Pettigrew S. Wright S. Pratt I. S. Blane S. Biagioni N. (2017). Potential in-class strategies to increase children's vegetable consumption. Public Health Nutr. 20, 1491–1499. 10.1017/S136898001700012X 28202096 PMC10261301

[B68] Sideli L. Coco G. L. Bonfanti R. C. Borsarini B. Fortunato L. Sechi C. . (2021). Effects of COVID-19 lockdown on eating disorders and obesity: a systematic review and meta-analysis. Eur. Eat. Disord. Rev. 29, 826–841. 10.1002/erv.2861 34460991 PMC8652707

[B69] Sim L. (2019). Our eating disorders blind spot: sex and ethnic/racial disparities in help-seeking for eating disorders. Mayo Clin. Proc. 94, 1398–1400. 10.1016/j.mayocp.2019.06.006 31378224

[B70] Simone M. Emery R. L. Hazzard V. M. Eisenberg M. E. Larson N. Neumark-Sztainer D. (2021). Disordered eating in a population-based sample of young adults during the COVID-19 outbreak. Int. J. Eat. Disord. 54, 1189–1201. 10.1002/eat.23505 33720460 PMC8250323

[B71] Springall G. Cheung M. Sawyer S. M. Yeo M. (2021). Impact of the coronavirus pandemic on anorexia nervosa and atypical anorexia nervosa presentations to an Australian tertiary paediatric hospital. J. Paediatr. Child Health. 58, 491–496. 10.1111/jpc.15755 34570958 PMC8661708

[B72] Stewart S. L. Toohey A. Celebre A. Poss J. W. (2021). Abuse, mental state, and health factors pre and during the COVID-19 pandemic: a comparison among clinically referred adolescents in Ontario, Canada. Int. J. Environ. Res. Public Health 18, 10184. 10.3390/ijerph181910184 34639487 PMC8507612

[B73] Striegel-Moore R. H. Rosselli F. Perrin N. DeBar L. Wilson G. T. May A. . (2009). Gender difference in the prevalence of eating disorder symptoms. Int. J. Eat. Disord. 42, 471–474. 10.1002/eat.20625 19107833 PMC2696560

[B74] Sun F. Zhu J. Tao H. Ma Y. Jin W. (2021). A systematic review involving 11,187 participants evaluating the impact of COVID-19 on anxiety and depression in pregnant women. J. Psychosomat. Obstetr. Gynecol. 42, 91–99. 10.1080/0167482X.2020.1857360 33327827

[B75] Tan E. J. Meyer D. Neill E. Phillipou A. Toh W. L. Van Rheenen T. E. . (2020). Considerations for assessing the impact of the COVID-19 pandemic on mental health in Australia. Aust. N. Z. J. Psychiatry 54, 1067–1071. 10.1177/0004867420947815 32746614 PMC7404085

[B76] Termorshuizen J. D. Watson H. J. Thornton L. M. Borg S. Flatt R. E. MacDermod C. M. . (2020). Early impact of COVID-19 on individuals with self-reported eating disorders: a survey of ~1,000 individuals in the United States and the Netherlands. Int. J. Eat. Disord. 53, 1780–1790. 10.1002/eat.23353 32720399

[B77] Toye F. Seers K. Allcock N. Briggs M. Carr E. Barker K. (2014). Meta-ethnography 25 years on: challenges and insights for synthesising a large number of qualitative studies. BMC Med. Res. Methodol. 14, 80. 10.1186/1471-2288-14-80 24951054 PMC4127190

[B78] Troop N. A. Holbrey A. Treasure J. L. (1998). Stress, coping, and crisis support in eating disorders. Int. J. Eat. Disord. 24, 157–166. 10.1002/(SICI)1098-108X(199809)24:2<157::AID-EAT5>3.0.CO;2-D 9697014

[B79] Wang C. Wen W. Zhang H. Ni J. Jiang J. Cheng Y. . (2021). Anxiety, depression, and stress prevalence among college students during the COVID-19 pandemic: a systematic review and meta-analysis. J. Am. Coll. Health 1–8. 10.1080/07448481.2021.1960849 34469261

[B80] Wang L. Chen L. Jia F. Shi X. Zhang Y. Li F. . (2021). Risk factors and prediction nomogram model for psychosocial and behavioural problems among children and adolescents during the COVID-19 pandemic: a national multicentre study Risk Factors of Childhood Psychosocial Problems. J. Affect. Disord. 294, 128–136. 10.1016/j.jad.2021.06.077 34284318 PMC8433597

[B81] Wong E. Mavondo F. Fisher J. (2020). Patient feedback to improve quality of patient-centred care in public hospitals: a systematic review of the evidence. BMC Health Serv. Res. 20, 530. 10.1186/s12913-020-05383-3 32527314 PMC7291559

[B82] Zeiler M. Wittek T. Kahlenberg L. Gröbner E.-M. Nitsch M. Wagner G. . (2021). Impact of COVID-19 confinement on adolescent patients with anorexia nervosa: a qualitative interview study involving adolescents and parents. Int. J. Environ. Res. Public Health 18, 4251. 10.3390/ijerph18084251 33923791 PMC8074137

