# Supplementary material for: The impacts of COVID-19 on eating disorders and disordered eating: A mixed studies systematic review and implications
Source: Front Psychol. 2022 Sep 6;13:926709. doi: 10.3389/fpsyg.2022.926709 (PMC9487416; doi:10.3389/fpsyg.2022.926709)
Supplement: Supplementary file 1 [file Data_Sheet_1.docx]

**Supplementary Information**

| Table 1. Mixed Methods Appraisal Tool (MMAT) Rating of Methodological Quality | | | | | | |
| --- | --- | --- | --- | --- | --- | --- |
| Qualitative | | | | | | |
|  | Is the qualitative approach appropriate to answer the research question? | Are the qualitative data collection methods adequate to address the research question? | Are the findings adequately derived from the data? | Is the interpretation of results sufficiently substantiated by data? | Is there coherence between qualitative data sources, collection, analysis, and interpretation? | Rating |
| Brown [1] | Yes | Yes | Yes | Yes | Yes | 5/5 |
| Clark Bryan [2] | Yes | Yes | Yes | Yes | Yes | 5/5 |
| Frayn, Fojtu and Juarascio [3] | Yes | Yes | Yes | Yes | Yes | 5/5 |
| Hunter and Gibson [4] | Yes | Yes | Yes | Yes | Yes | 5/5 |
| Nutley [5] | Yes | Yes | Yes | Yes | Yes | 5/5 |
| Zeiler [6] | Yes | Yes | Yes | Yes | Yes | 5/5 |
| Quantitative Randomized Control | | | | | | |
|  | Is randomization appropriately performed? | Are the groups comparable at baseline? | Are there complete outcome data? | Are outcome assessors blinded to the intervention provided? | Did the participants adhere to the assigned intervention? | Rating |
| Zhou and Wade [7] | No | Yes | Yes | No | Yes | 3/5 |
| Quantitative Non-Randomised Control | | | | | | |
|  | Are the participants representative of the target population? | Are measurements appropriate regarding both the outcome and intervention (or exposure)? | Are there complete outcome data? | Are the confounders accounted for in the design and analysis? | During the study period, is the intervention administered (or exposure occurred) as intended? | Rating |
| Albert [8] | Yes | No | Yes | Can’t tell | Yes | 3/5 |
| Ayton [9] | Yes | Yes | Yes | Can’t tell | Yes | 4/5 |
| Baceviciene and Jankauskiene [10] | Yes | Yes | Can’t tell | Can’t tell | Yes | 3/5 |
| Baenas [11] | Yes | Yes | Yes | Can’t tell | Yes | 4/5 |
| Beghi [12] | Yes | Can’t tell | Yes | No | Yes | 3/5 |
| Breiner, Miller and Hormes [13] | Yes | Yes | Yes | Can’t tell | Yes | 4/5 |
| Calugi [14] | Yes | Yes | Yes | Yes | Yes | 5/5 |
| Castellini [15] | Yes | No | Can’t tell | Yes | Yes | 3/5 |
| Cecchetto [16] | Can’t tell | No | Yes | Can’t tell | Yes | 2/5 |
| Chadi [17] | Yes | Yes | Can’t tell | Can’t tell | Yes | 3/5 |
| Chen [18] | Yes | Yes | Can’t tell | No | Yes | 3/5 |
| Christensen [19] | Can’t tell | Yes | Can’t tell | Yes | Yes | 3/5 |
| Coimbra, Paixão and Ferreira [20] | No | Yes | Can’t tell | No | Yes | 2/5 |
| Conceição [21] | Yes | Yes | Can’t tell | Yes | Yes | 4/5 |
| Giel [22] | Can’t tell | Yes | Can’t tell | Can’t tell | Yes | 2/5 |
| Horita, Nishio and Yamamoto [23] | Yes | No | Can’t tell | Can’t tell | Yes | 2/5 |
| Karakose, Yirci, Basyigit and Kucukcakir [24] | Yes | No | Can’t tell | Can’t tell | Yes | 2/5 |
| Keel [25] | No | Yes | Yes | Yes | Yes | 4/5 |
| Kim [26] | Yes | Yes | Yes | Yes | Yes | 5/5 |
| Koenig [27] | Yes | Can’t tell | Can’t tell | Yes | Yes | 3/5 |
| Kohls [28] | Yes | Yes | Yes | Yes | Yes | 5/5 |
| Lin [29] | Yes | Yes | Yes | Yes | Yes | 5/5 |
| Martínez-de-Quel, Suárez-Iglesias, López-Flores and Pérez [30] | Yes | No | Yes | Can’t tell | Yes | 3/5 |
| Matthews, Kramer, Peterson and Mitan [31] | Yes | Yes | Yes | Can’t tell | Yes | 4/5 |
| Meda [32] | Yes | No | Can’t tell | No | Yes | 2/5 |
| Monteleone [33] | Can’t tell | No | Yes | No | Yes | 2/5 |
| Monteleone [34] | Yes | No | Yes | Yes | Yes | 4/5 |
| Otto [35] | Yes | Yes | Yes | Can’t tell | Yes | 4/5 |
| Phelan, Behan and Owens [36] | Yes | No | Can’t tell | No | Yes | 2/5 |
| Puhl [37] | Yes | Yes | No | Yes | Yes | 4/5 |
| Springall, Cheung, Sawyer and Yeo [38] | Yes | Yes | Yes | Yes | Yes | 5/5 |
| Stewart, Toohey, Celebre and Poss [39] | Yes | Yes | Yes | Yes | Yes | 5/5 |
| Thompson and Bardone-Cone [40] | Yes | Yes | Yes | Yes | Yes | 5/5 |
| Trott [41] | Can’t tell | Can’t tell | Can’t tell | No | Yes | 1/5 |
| Wang [42] | Yes | Yes | Yes | Yes | Yes | 5/5 |
| Vaccaro [43] | Yes | Yes | Yes | No | Yes | 4/5 |
| Quantitative Descriptive | | | | | | |
|  | Is the sampling strategy relevant to address the research question? | Is the sample representative of the target population? | Are the measurements appropriate? | Is the risk of nonresponse bias low? | Is the statistical analysis appropriate to answer the research question? | Rating |
| Athanasiadis [44] | Yes | Yes | Yes | No | Yes | 4/5 |
| Carcavilla [45] | Yes | Yes | No | Can’t tell | Can’t tell | 2/5 |
| Czepczor-Bernat, Swami, Modrzejewska and Modrzejewska [46] | Yes | Yes | Yes | Can’t tell | Yes | 4/5 |
| Dale [47] | Yes | Yes | Yes | Can’t tell | Yes | 4/5 |
| Elmacıoğlu [48] | Yes | Can’t tell | No | Can’t tell | Yes | 2/5 |
| Favreau [49] | Yes | Can’t tell | Yes | Can’t tell | Yes | 3/5 |
| Flaudias [50] | Yes | Yes | No | No | Yes | 3/5 |
| Flaudias [51] | Yes | Yes | No | No | Yes | 3/5 |
| Gholmie [52] | Yes | Yes | Yes | Yes | Yes | 5/5 |
| Jordan [53] | Yes | Yes | Yes | Can’t tell | Yes | 4/5 |
| Leenaerts, Vaessen, Ceccarini and Vrieze [54] | Can’t tell | Can’t tell | No | Yes | Yes | 2/5 |
| Papandreou [55] | Can’t tell | No | Yes | Can’t tell | Yes | 2/5 |
| Phillipou [56] | Yes | Yes | Yes | Can’t tell | Yes | 4/5 |
| Pourrazi, Modaberi and Kabiri [57] | Can’t tell | Can’t tell | Yes | Can’t tell | Yes | 2/5 |
| Ramalho [58] | Can’t tell | Can’t tell | Yes | Can’t tell | Yes | 2/5 |
| Scharmer [59] | Can’t tell | Yes | Yes | Can’t tell | Yes | 3/5 |
| Schlegl, Maier, Meule and Voderholzer [60] | Can’t tell | Yes | Yes | Yes | Yes | 4/5 |
| Schlegl, Meule, Favreau and Voderholzer [61] | Yes | Yes | Yes | Yes | Yes | 5/5 |
| Serin and Koç [62] | Yes | Yes | Yes | Can’t tell | Yes | 4/5 |
| Termorshuizen [63] | Yes | Yes | Yes | Can’t tell | Yes | 4/5 |
| Wang [64] | Can’t tell | Yes | Yes | Yes | Yes | 4/5 |
| Mixed Methods | | | | | | |
|  | Is there an adequate rationale for using a mixed methods design to address the research question? | Are the different components of the study effectively integrated to answer the research question? | Are the outputs of the integration of qualitative and quantitative components adequately interpreted? | Are divergences and inconsistencies between quantitative and qualitative results adequately addressed? | Do the different components of the study adhere to the quality criteria of each tradition of the methods involved? | Rating |
| Branley-Bell and Talbot [65] | Yes | Yes | Yes | Yes | Can’t tell | 4/5 |
| Brownstone [66] | Can’t tell | Yes | no | No | Yes | 2/5 |
| McCombie [67] | Can’t tell | Yes | No | No | Yes | 1/5 |
| Muzi, Sansò and Pace [68] | Yes | Yes | No | Can’t tell | Can’t tell | 2/5 |
| Phillipou [69] | Yes | Yes | Yes | Yes | Yes | 5/5 |
| Raykos [70] | Yes | Yes | Yes | Yes | Can’t tell | 4/5 |
| Richardson, Patton, Phillips and Paslakis [71] | Yes | Yes | Yes | Can’t tell | Yes | 4/5 |
| Simone [72] | Yes | Yes | Yes | Yes | Yes | 4/5 |

| Table 2. Main Themes and Sub-themes of Included Qualitative and Mixed Methods Studies | |
| --- | --- |
| **Qualitative Studies** | |
| **Brown [1]** | 1. **Social restrictions** 2. Social isolation 3. Changes in accountability 4. Increased responsibility 5. **Functional restrictions** 6. Lack of routine and structure 7. A need for intentionality 8. Secrecy 9. **Restriction in accessing professional support** 10. Accessibility of support |
| **Clark Bryan [2]** | **Patients**   1. **Reduced access to ED services** 2. Disparity in access to ED services 3. Reliance on remove support from professionals 4. Premature discharge from services 5. **Disruption to routine and activities in the community** 6. Coping with changing routine and structure 7. Disrupted transitions into community living 8. Reduced motivations for recovery 9. **Heightened psychological distress and ED symptoms** 10. Concerns over access to food and focus on exercise 11. Increased fear and anxiety 12. **Increased attempts at self-management in recovery** 13. Experiences of increased self-efficacy 14. Seeking alternative practical coping strategies and resources   **Carers**   1. **Concern over provision of professional support for patients** 2. Fears over premature discharge of patients from services 3. Change in delivery of support for patients 4. **Increased practical demands placed on carers in lockdown** 5. Managing patient and family needs in lockdown 6. Curtailment of normal activities and lack of routine 7. Challenges around shielding and social distancing 8. **Managing new challenges around patient wellbeing** 9. Increased displays of anxiety 10. Reporting new food-related triggers 11. Spotting signs of AN relapse 12. **New Opportunities** 13. Gratitude for increased time at home 14. Noticing increased self-efficacy 15. Utilizing adaptive perspectives and approaches |
| **Frayn [3]** | 1. **Impact of COVID-19 on eating disorder symptoms** 2. Variability in the improvement or exacerbation of symptoms due to COVID-19 3. Changes in the physical environment were associated with symptom improvement 4. Social implications of COVID-19 were associated with both symptom improvement and deterioration 5. Greater overall stress/anxiety levels leads to more binge episodes 6. **Perceptions of tele-therapy** 7. Tele-therapy was positively perceived by the majority of participants 8. Tele-therapy is convenient and facilitates attendance and engagement 9. Tele-therapy makes treatment accessible for those who would be otherwise unable to attend 10. Tele-therapy is perceived as more impersonal than in-person therapy 11. Tele-therapy may be hindered by logistical or technical concerns 12. **Addressing COVID-19 in the present treatment** 13. Consistent or increased motivation to participant in treatment 14. Variable desire for COVID-related concerns to be addressed in ED treatment 15. Conflicts between COVID-19 and treatment |
| **Hunter and Gibson [4]** | 1. **Loss on control** 2. Difficulties accessing ‘safe’ foods 3. Increase in anxiety and stress 4. Exacerbations of negative disordered thoughts and behaviours 5. **Support during confinement** 6. Increased reliance on family and friends as support networks 7. Accessing services and professional support during COVID-19 8. Risks of tele-health 9. **Time of reflection on recovery** |
| **Nutley [5]** | 1. **Change in ED symptoms** 2. Increased ED symptomatology 3. Decreased ED symptomatology 4. Negative body image 5. **Change in exercise routine** 6. Changes in exercise behaviour 7. Exercise facilities closed or inaccessible 8. **Impact of quarantine on daily life** 9. Change in routine/environment 10. Food hoarding or shortages 11. Navigating triggering relationships 12. **Emotional well-being** 13. Negative affect 14. **Help-seeking behaviour** 15. Willingness to recover 16. Currently receiving treatment 17. Unable to receive treatment 18. Requesting advice or accountability from other reddit users 19. Words of encouragement 20. Seeking help on behalf of another individual 21. **Associated risks and health outcomes** 22. Substance use behaviour 23. Adverse health outcomes |
| **Zeiler [6]** | **Patients**   1. **Restrictions of personal freedom** 2. Feeling of being imprisoned and bored 3. Tensions between patients and family members 4. Less motivation to work on recovery 5. Missing close others 6. **Interruption of the treatment routine** 7. Risks through self-monitored weight 8. Challenges and opportunities of teletherapy 9. Changes of staff cohorts and treatment offers in the inpatient setting 10. **Changes in the eating disorder and other psychopathology** 11. Boredom and feeling of being observed triggering eating disorder symptoms 12. COVID-19-related fears and compulsions 13. Improvement in symptoms indicating a normal treatment course 14. **Opportunities of the COVID-19 period** 15. Less stress allows for paying more attention to own needs 16. Experiencing a more intensive time with the family 17. Promoting autonomy and self-organization skills   **Parents**   1. **Changes in the daily routines** 2. Challenges regarding the organization and creation of day structure 3. Experiencing more time with family 4. Slowing down for the whole family 5. **Parents’ perspective regarding the outpatient and inpatient treatment** 6. Reservations about reduced outpatient monitoring and increased teletherapy 7. Altered family contact with the child during the inpatient treatment 8. Maintaining contact between parents and treatment staff 9. **Challenges and benefits of the COVID-19 confinement for the eating disorder symptoms and mental health of the child** |
| **Mixed Methods Studies** | |
| **Branley-Bell and Talbot [65]** | 1. **ED behaviours as an ‘auxiliary control mechanism’** 2. **Loss of auxiliary control after lockdown** |
| **Brownstone [66]** | 1. **Losing affirming spaces and security** 2. Affirming spaces and practices related to TGNB identity 3. Work and financial insecurity connected to disordered eating 4. **Gaining affirming and supportive spaces online** 5. **Reflecting on embodied gender and identities** 6. **Realising new insights and connections** 7. Discovery to TGNB gender identity 8. Connections made between gender dysphoria and disordered eating 9. New insights about eating and recovery 10. **Considering the self in social context** 11. Boundary setting and deepened relationships 12. Activation and empathy around social justice |
| **McCombie [67]** | 1. **Mechanisms contributing to ED exacerbation** 2. Isolation 3. Worry, rumination, or worsening anxiety and depression 4. Media impact 5. Structure and routine 6. **Positive aspects of life in lockdown** |
| **Muzi [68]** | Not applicable* |
| **Phillipou [69]** | **ED Group**   1. **Impact of the pandemic on mental health** 2. **Re-triggering of eating disorder** 3. **Social isolation** 4. **Anxiety and fear around family and the economy** 5. **Employment**   **Non-ED Group**   1. **Social Isolation** 2. **Anxiety** 3. **Financial and economic impacts of the pandemic** 4. **Concerns about travel** |
| **Raykos [70]** | Not applicable* |
| **Richardson [71]** | 1. **Lack of access to treatment** 2. **Worsening of ED symptoms** 3. **Feeling out of control** 4. **Need for support** |
| **Simone [72]** | 1. **Mindless eating and snacking** 2. **Increase food consumption** 3. **Generalised decrease in appetite or dietary intake** 4. **Eating to cope** 5. **Pandemic-related reductions in dietary intake** 6. **Re-emergence or marked increase in eating disorders symptoms** |
| ***Note*.** * study whereby qualitative methods were used (e.g., Eating Disorder Examination Interview) but no themes were generated. | |
