# Supplementary material for: The impacts of COVID-19 on eating disorders and disordered eating: A mixed studies systematic review and implications
Source: Front Psychol. 2022 Sep 6;13:926709. doi: 10.3389/fpsyg.2022.926709 (PMC9487416; doi:10.3389/fpsyg.2022.926709)
Supplement: Supplementary file 2 [file Table_1.docx]

| Table 1. Characteristics of included studies and their findings | | | | | | | | | | | | | |
| --- | --- | --- | --- | --- | --- | --- | --- | --- | --- | --- | --- | --- | --- |
| Author (date) | Country | Study Design | Study Population | Sample Size | ED Clinical Status | Gender Distribution (% Female) | Age Distribution (*M*(*SD*)) | Ethnicity / Race (% White) | SES | Minority / Understudied Group | ED Measure of Interest | Main Findings and Effect Size (if available) | MMAT |
| Albert et al. (2021) | Italy | Cross-sectional | Bariatric surgery waiting list cohort | 56 | No | 67.9% | 47.9 (9.3) | NS | NS | No | Questions assessing maladaptive eating behaviours | No significant change in the portion of individuals showing binge eating behaviours before or after lockdown. | Low |
| Athanasiadis et al. (2021) | United States | Cross-sectional | Postoperative bariatric patients | 208 | No | 86.0% | 48.9 (11.2) | 86.1% | NS | No | Items derived from ELCS | 48.2% of respondents reported increased loss of control when eating and 19.5% reported increased binge eating compared to before the pandemic. | Med |
| Ayton et al. (2021) | United Kingdom | Longitudinal | ED patients | 351 referrals | Yes | 97.0% | 29.6 (11.0) | NS | NS | No | DSM-5 | Absolute number of referrals increased by 21% during the pandemic. | Med |
| Baceviciene and Jankauskiene (2021) | Lithuania | Pre- and post-lockdown observational study | University students | 230 | No | 79.1% | 23.9 (5.4) | NS | NS | No | EDE-Q | No significant differences in disordered eating scores in men or women before or during lockdown. | Low |
| Baenas et al. (2020) | Spain | Cross-sectional | ED patients | 74 | Yes | 95.9% | 32.1 (12.8) | NS | NS | No | DSM-5 | No significant differences in worsening of ED symptoms and COVID-related concerns. | Med |
| Beghi et al. (2021) | Italy | Longitudinal observational | ED patients | NS | Yes | Lockdown = 43.4%, Control = 55.8% | NS | Lockdown = 86.6% Italian, Control = 80.2% Italian | NS | No | NS | Lockdown was associated with a 14.5% decrease in psychiatric assessments and 16.8% decrease in individuals observed compared to 2019. Lockdown was associated with significantly higher psychiatric ward admissions. | Low |
| Branley-Bell and Talbot (2021) | United Kingdom | Mixed methods | Individuals with self-reported ED experience | 58 | Yes | 98.3% | 30.9 (11.1) | NS | NS | No | NS | Most participants reported no change in their ED status at the beginning of lockdown compared to the easing of restrictions. Participants reported two main themes: ED behaviours acted as an “auxiliary control mechanical” and a loss of auxiliary control after lockdown. | Med |
| Author (date) | Country | Study Design | Study Population | Sample Size | ED Clinical Status | Gender Distribution (% Female) | Age Distribution (*M*(*SD*)) | Ethnicity / Race (% White) | SES | Minority / Understudied Group | ED Measure of Interest | Main Findings and Effect Size (if available) | MMAT |
| Breiner et al. (2021) | United States | Cross-sectional | Community sample | 159 | No | 90.6% | 27.6 (11.7) | 90.6% | 78.7% university degree or higher | No | EDE-Q | No significant change in EDE-Q scores between those with and without a previous ED diagnosis during the pandemic. No significant differences in EDE-Q scores before or during the pandemic. | Med |
| Brown et al. (2021) | United Kingdom | Qualitative Interview | ED patients | 10 | Yes | 90% | 29.6 | 100% | NS | No | Self-reported ED diagnosis | Patients reported three main themes: social restrictions, functional restrictions, and restrictions in access to professional support. Impact of lockdown was described as a catalyst for DE behaviours or an effort to recover. | High |
| Brownstone et al. (2021) | United States | Mixed methods | Community sample | 13 | No | 7.7% | NS | 61.5% | NS | Transgender and gender nonbinary individuals | EDE-Q | Most participants reported increased DE, while half reported increased binge eating behaviours and body dissatisfaction compared to before the pandemic. This was supported by EDE-Q responses. | Low |
| Calugi et al. (2021) | Italy | Retrospective case-control study | Patients with severe obesity | 258 | No | 69.8% | 57.0 (14.2) lockdown patients, 56.5 (14.0) control patients | NS | NS | No | EDE-Q | No significant differences in EDE-Q scores in patients experiencing the lockdown compared to controls. | High |
| Carcavilla et al. (2021) | Spain | Cross-sectional | Family caregiver of people with dementia | 106 | No | 74.5% | NS | NS | NS | No | NS | 12% of participant reported ED because of the pandemic. | Low |
| Castellini et al. (2020) | Italy | Longitudinal study | ED patients | 171 | Yes | 100.0% | Patients = 31.7 (12.8), control = 30.5 (10.9) | NS | NS | No | DSM-5, EDE-Q | ED patients reported a significant increase in binge eating (*p* < .05, *d* = 0.53) and compensatory physical exercises (*p* < .05, *d* = 0.39) during lockdown compared to controls. No significant differences in EDE-Q scores between groups pre- and during-lockdown. | Low |
| Author (date) | Country | Study Design | Study Population | Sample Size | ED Clinical Status | Gender Distribution (% Female) | Age Distribution (*M*(*SD*)) | Ethnicity / Race (% White) | SES | Minority / Understudied Group | ED Measure of Interest | Main Findings and Effect Size (if available) | MMAT |
| Cecchetto et al. (2021) | Italy | Longitudinal study | Community sample | 365 | No | 73.1% | 35.1 (13.6) | NS | NS | No | BEDS-7 | Compared to normative pre-pandemic data, binge eating was significantly higher during the pandemic, with lower scores reported as lockdown progressed. | Low |
| Chadi et al. (2021) | Canada | Retrospective study | Paediatric ED visitors | 154 visitors | Yes | NS | NS | NS | NS | Children and adolescents | NS | Total number of annual visits increased by 62% for EDs in 2020 compared to the mean for 2018-2019. | Low |
| Chen et al. (2020) | United Kingdom | Controlled interrupted time series study | Individuals seeking mental health services | NS | No | NS | NS | NS | NS | No | NS | No significant differences in referrals to the ED team per day during lockdown and 2019. | Low |
| Christensen et al. (2021) | United States | Cross-sectional | University students | 579 | No | 76.3% | 21.8 (5.3) | 84.1% | NS | No | CIA, EDDS | No significant differences in prevalence of ED diagnosis between the pre-COVID-19 and COVID-19 cohort. | Low |
| Clark Bryan et al. (2020) | United Kingdom | Qualitative interviews | ED patients and their carers | Patients = 21, carers = 28 | Yes | Patients = 85.7%, Carers = 82.1% | Patients = 25.5 (5.6), Carers = 54.0 (7.3) | NS | NS | No | DSM-5 | Patients reported four main themes: reduced access to ED services, disruption to routine, heightened psychological distress and ED symptoms, and increased attempts at self-management in recovery. Carers reported four main themes: concern for provision of professional support for patients, increased demands placed on carers, managing patient wellbeing, and new opportunities. | High |
| Coimbra et al. (2021) | Portugal | Cross-sectional | Women with different BMI | 580 | No | 100% | 26.9 (9.4) | NS | 14.8 (2.4) years of education | No | EDE-Q | Women in the obesity and pre-obesity group had significantly higher shape concerns, weight concerns, and binge eating during the pandemic. | Low |
| Conceição et al. (2021) | Portugal | Longitudinal design | Bariatric surgery patients | 101 | No | COVID group = 94.3%, nonCOVID | COVID group = 50.8 (12.4), nonCOVID | NS | COVID group = 22.9% university educated, | No | EDE-Q | Patients who experienced their 3-year surgery follow up during lockdown had significantly higher weight concern (*p* = .005, ƞ^2^p = .05), | Med |
| Author (date) | Country | Study Design | Study Population | Sample Size | ED Clinical Status | Gender Distribution (% Female) | Age Distribution (*M*(*SD*)) | Ethnicity / Race (% White) | SES | Minority / Understudied Group | ED Measure of Interest | Main Findings and Effect Size (if available) | MMAT |
|  |  |  |  |  |  | group = 83.3% | group = 50.1 (10.7) |  | nonCOVID group = 19.7% university educated |  |  | but not shape concern, restriction, or food concerns compared to patients who had their 3-year follow up prior to lockdown. |  |
| Czepczor-Bernat et al. (2021) | Poland | Cross-sectional | Community sample | 671 | No | 100% | 32.5 (11.4) | 99.1% | 64.7% had completed a university degree or above | No | EDI | Significant ED differences across stress and weigh groups (p < .001, n_p_  ^2^ = 0.05 - 0.14), with individuals with high COVID-related stress having higher levels of DE compared to those with low COVID-related stress. | Med |
| Dale et al. (2021) | Austria | Cross-sectional | Apprentices | 1442 | No | 53.5% | 18.2 (2.3) | NS | NS | No | EAT-8 | 50.6% of the total sample met cut-off for DE behaviour during the pandemic. | Med |
| Elmacıoğlu et al. (2021) | Turkey | Cross-sectional | Community sample | 1036 | No | 79.8% | 33.1 (13.0) | NS | 74.8% had completed a university degree or above | No | Revised TFEQ-18 | Pandemic lead to an increase in uncontrolled eating behaviours, but not cognitive restriction behaviours | Low |
| Favreau et al. (2021) | Germany | Cross-sectional | Inpatients with various psychiatric disorders | 538 (AN = 88, BN = 30) | Yes | 70.3% | NS | NS | NS | No | NS | 35.3% of AN patients and 23.3% of BN patients strongly agreed that their symptoms worsened due to the pandemic. | Low |
| Flaudias et al. (2020) | France | Cross-sectional | University students | 5,738 | No | 74.6% | 21.2 (4.5) | NS | NS | No | EDI-2 | Higher stress related to the lockdown was associated with higher likelihood of reporting binge eating (*p* = .004, OR = 1.12) and dietary restriction (*p* < .001, OR 1.17) over the past week. | Low |
| Flaudias et al. (2021) | France | Cross-sectional | University student | 5,738 | No | 74.6% | 21.2 (4.5) | NS | NS | No | EDI-2, SCOFF | Compared to those with low stress associated with lockdown, mildly and highly stressed students reported more restricted eating (95% CI, 0.2 to 0.44, *p* < 0.001; 95% CI, 0.45 to 0.67, *p* < 0.001) and compulsive eating (95% CI, 0.48 to 0.79, *p* < 0.001; 0.85 to 1.17, *p* < 0.001) in the last week. | Low |
| Author (date) | Country | Study Design | Study Population | Sample Size | ED Clinical Status | Gender Distribution (% Female) | Age Distribution (*M*(*SD*)) | Ethnicity / Race (% White) | SES | Minority / Understudied Group | ED Measure of Interest | Main Findings and Effect Size (if available) | MMAT |
| Frayn et al. (2021) | United States | Qualitative interviews | Binge eating spectrum disorder patients | 11 | Yes | 63.6% | 42.8 (14.2) | 81.8% | 45.5% has household income over 100,000 | No | NS | Patients reported four main themes: variability in the improvement or exacerbation of symptoms, changes in the physical environment associated with improvement of symptoms, social implications of the pandemic associated with symptom improvement and deterioration, and greater stress and anxiety leading to more binge episodes. | High |
| Gholmie (2021) | United States | Cross-sectional | Individuals with celiac disease | 50 | No | 70.0% | 29.6 (7.4) | 94.0% | 62.0% had a household income of >$100,000 | No | EPSI | Binge eating and restricting was significantly lower during the pandemic compared to before the pandemic. No significant differences in the other EPSI subscales before or during the pandemic. | High |
| Giel et al. (2021) | Germany | Qualitative interview | Binge ED patients | 42 | Yes | 90.9% | Baseline = 41.1 (12.6), End of treatment = 41.3 (12.6), COVID follow-up = 45.5 (12.6) | NS | NS | No | DSM-5, EDE | Significant increase in binge eating episodes for the four weeks during lockdown compared to before the pandemic (Wald‐*^χ^*^2^ = 15.22; corrected *p* < 0.001). Self-reported ED symptoms at lockdown follow up were significantly higher than baseline and end of treatment (Wald‐ *x^2^* = 7.51; corrected *p* = 0.006; Wald‐*x^2^* = 35.52; corrected *p* < 0.001). | Low |
| Horita et al. (2021) | Japan | Cross-sectional | University students | 2020 cohort = 766, 2019 cohort = 400 | No | 2020 cohort = 45.3%, 2019 cohort = 56.5% | NS | NS | NS | No | K10 | Significant differences in eating concerns between 2020 or 2019 cohorts. | Low |
| Hunter and Gibson (2021) | United Kingdom | Qualitative interview | People with anorexia nervosa | 12 | Yes | 91.7% | 31.8 | NS | NS | No | NS | Participants reported three main themes: loss of control, support during confinement, and time of reflection on recovery. | High |
| Author (date) | Country | Study Design | Study Population | Sample Size | ED Clinical Status | Gender Distribution (% Female) | Age Distribution (*M*(*SD*)) | Ethnicity / Race (% White) | SES | Minority / Understudied Group | ED Measure of Interest | Main Findings and Effect Size (if available) | MMAT |
| Jordan et al. (2021) | United States | Cross-sectional | Caregivers of children | 140 | No | 88.6% | 39.8 (6.9) | 88.4% | NS | No | EDE-Q-Short Form | Stress during the pandemic was positively associated with greater DE. | Med |
| Karakose et al. (2021) | Turkey | Cross-sectional | School administrators | 266 | No | 13.2% | NS | NS | 16.2% has received a graduate- level education | No | DEBQ | COVID-19 fear was positively associated with external eating behaviour and restrained eating behaviour. | Low |
| Keel et al. (2020) | United States | Longitudinal design | University students | 90 | No | 88.0% | 19.5 (1.3) | 78.0% | NS | No | EDDS | Participants reported higher perceived changes in weight and shape concerns (*p* < 0.001, *d* = 0.93) since the beginning of the pandemic. | Med |
| Kim et al. (2021) | United States | Case-control | University students | 8,613 | No | Pre-pandemic = 73.1%, pandemic = 69.7% | Pre-pandemic = 18.9 (2.1), Pandemic = 19.5 (3.3) | Pre-pandemic = 72.0%, Pandemic = 74.8% | NS | No | SWED | Significantly greater proportion of participants meeting criteria for bulimia nervosa/binge ED, compared to pre-pandemic times (*p* < 0.001, OR 1.54, 95% CI 1.28, 1.85) | High |
| Koenig et al. (2021) | Germany | Matched convenience design | Adolescents | 648 | No | Pre-lockdown = 69.1%, post-lockdown = 69.4% | 14.9 (1.9) | NS | NS | Children and adolescents | WCS, EDE-Q | No significant differences in DE pre-lockdown or post-lockdown | Low |
| Kohls et al. (2021) | Germany | Cross-sectional | University students | 3,382 | No | 70.2% | 24.0 (4.7) | NS | NS | No | SEED | Higher perceived stress associated with the pandemic significantly predicted higher severity of bulimia nervosa related symptoms. | High |
| Leenaerts et al. (2021) | Belgium | Cross-sectional | Patients with bulimia nervosa | 15 | Yes | 100% | 23.0 | 87.0% European | Mean 15.0 years formal education | No | If participants experienced a loss of control over their eating since last assessment. | No significant differences in binge eating during the lockdown than before the lockdown. | Low |
| Lin et al. (2021) | United States | Retrospective analysis | Adolescents / young people | NS | Yes | NS | NS | NS | NS | Children and adolescents | NS | Increase in inpatient admissions, hospital bed-days, and outpatient care-related inquiries post-, compared to pre-pandemic. Outpatient ED assessments decreased at the onset and gradually increased during the pandemic. | High |
| Author (date) | Country | Study Design | Study Population | Sample Size | ED Clinical Status | Gender Distribution (% Female) | Age Distribution (*M*(*SD*)) | Ethnicity / Race (% White) | SES | Minority / Understudied Group | ED Measure of Interest | Main Findings and Effect Size (if available) | MMAT |
| Martínez-de-Quel et al. (2021) | Spain | Longitudinal | Community and university student sample | 161 | No | 37.0% | 35.0 (11.2) | NS | NS | No | EAT-26 | No significant differences in ED risk before or during lockdown. | Med |
| Matthews et al. (2021) | United States | Retrospective chart review | Young people with acute medical complications of anorexia nervosa or atypical anorexia nervosa | 163 | Yes | 82.8% | 15.2 (1.8) | 92.6% | NS | Children and adolescents | NS | Significant association between the pandemic period and readmissions within 30 days, with patients 8.7 times more likely to be readmitted to hospital within 30 days post-lockdown, than before lockdown. No significant differences in daily or monthly admissions pre- or post-lockdown. 1/3 of patients said the pandemic was a primary correlate to their ED. | Med |
| McCombie et al. (2020) | United Kingdom | Mixed methods | Individuals with lifetime EDs | 32 | Yes | 93.6% | 35.2 (10.3) | 100.0% | NS | No | EDE-Q | 88% of participants reported an exacerbation of ED symptoms due to lockdown. Participants reported two | Low |
|  |  |  |  |  |  |  |  |  |  |  |  | main themes: mechanisms that contributed to ED exacerbation including isolation, worry and anxiety, and routine, and positive aspects to life in lockdown. |  |
| Meda et al. (2021) | Italy | Cohort study | University students | 358 | Yes | 79.9% | 21.3 (2.1) | NS | NS | No | EDI-3 | No significant difference in DE during or after lockdown. Individuals with ED history had significantly higher ED risk scores after lockdown | Low |
| Monteleone, Cascino, et al. (2021) | Italy | Retrospective analysis | ED patients | 312 | Yes | 96.2% | 29.2 (12.1) | NS | NS | No | Adapted EDI-2, DSM-5 | Risk factors to ED symptoms significantly worsened between lockdown than pre-pandemic. Risk factor worsening was not influenced by ED diagnosis. | Low |
| Monteleone, Marciello, et al. (2021) | Italy | Retrospective analysis | ED patients | 312 | Yes | 96.2% | 29.2 (12.1) | NS | NS | No | Adapted EDI-2, DSM-5 | Purging, body dissatisfaction, binge eating, and self- induced vomiting were higher during lockdown than pre- or post-lockdown. No significant difference in weight concern. | Med |
| Author (date) | Country | Study Design | Study Population | Sample Size | ED Clinical Status | Gender Distribution (% Female) | Age Distribution (*M*(*SD*)) | Ethnicity / Race (% White) | SES | Minority / Understudied Group | ED Measure of Interest | Main Findings and Effect Size (if available) | MMAT |
| Muzi et al. (2021) | Italy | Mixed methods | Adolescents | 62 | No | 63.0% | 15.4 (1.7) | NS | 99% of participants parents had a job | Children and adolescents | BES | No significant differences in binge eating during the pandemic or pre-pandemic. | Low |
| Nutley et al. (2021) | United States | Qualitative analysis | Reddit users | 33 users | NS | NS | NS | NS | NS | NS | NS | Six themes were identified: change in ED symptoms, change exercise behaviours, impact of quarantine, emotional well-being, help-seeking behaviour, and risks and health outcomes. Majority of users indicted the pandemic negatively impacted their ED symptoms. | High |
| Otto et al. (2021) | United States | Retrospective chart review | ED patients | 248 | Yes | Pre-pandemic = 88.4%, during-pandemic = 90.2% | Pre-pandemic = 15.1 (2.8), during-pandemic = 15.2 (2.5) | Pre-pandemic = 84.9% during-pandemic = 90.2% | NS | Children and adolescents | NS | There was a 123% increase in ED admissions between April 202 and March 2021 compared to the same time for the previous three years. | Med |
| Papandreou et al. (2020) | Spain | Cross-sectional | Community sample from Spain and Greece | 1,841 (1,002 in Spain, 839 in Greece) | No | Spain = 70.3%, Greece = 66.7% | Spain = 46.1 (13.3), Greece = 42.4 (11.7) | NS | NS | No | DEBQ | Significantly lower restraint and external eating behaviours in participants living in Spain with stricter lockdown measures, compared to participants in Greece. | Low |
| Phelan et al. (2021) | Ireland | Observational study | Women of reproductive age | 1,031 | No | 100% | 36.7 (6.6) | 97.0% | 68% work full time | No | “Have you suffered from binge eating before/during the COVID-19 pandemic?” | Significant increase in binge eating during the pandemic compared pre-pandemic. | Low |
| Phillipou et al. (2020) | Australia | Cross-sectional | Community sample | 5,469 (180 with self-reported ED history) | Yes | ED group = 95.6%, non-ED group = 80.0% | ED group = 30.5 (8.2), non-ED group = 40.6 (13.7) | NS | NS | No | EDE-Q | Most of the ED group reported increased food restrictions (67%), and most reported no differences in purging (77%) or binge eating (69%). In the non-ED group, most reported no difference in restricting (59%), binge eating (60%), and purging (98%). | Med |
| Author (date) | Country | Study Design | Study Population | Sample Size | ED Clinical Status | Gender Distribution (% Female) | Age Distribution (*M*(*SD*)) | Ethnicity / Race (% White) | SES | Minority / Understudied Group | ED Measure of Interest | Main Findings and Effect Size (if available) | MMAT |
| Phillipou et al. (2021) | Australia | Mixed methods | Community sample | 4,915 (231 with self-reported ED history) | Yes | ED group = 94.4%, non-ED group = 78.9% | ED group = 28.7 (7.4), non-ED group = 38.3 (14.2) | NS | NS | No | EDE-Q | Significant increase in restricting during the second wave. Binge eating, purging, and exercise behaviours did not differ between waves. ED group reported lower quality of life during lockdown compared to non-ED group. | High |
| Pourrazi et al. (2021) | Iran | Cross-sectional | Community sample | 705 | No | 71.2% | 29.7 (10.7) | NS | NS | No | EAT-26 | Significant increase in DE in women, but not men, after the outbreak. | Low |
| Puhl et al. (2020) | United States | Longitudinal cohort design | Community sample | 584 | No | 64.2% | 21.9 (2.0) | 30.2% | 74.1% had an SES of middle to high | No | EAT | Individuals who experienced weight stigma prior to the pandemic had increased likelihood of binge eating during the pandemic (OR = 2.88, *p* < .001). | Med |
| Ramalho et al. (2021) | Portugal | Cross-sectional | Community sample | 254 | No | 82.7% | 35.8 (11.8) | NS | 87.0% had a university degree or higher | No | DE Behaviours Screening Questionnaire, TFEQ | COVID-19 psychosocial impact was significantly associated with uncontrolled eating but not cognitive restraint. Full mediation between COVID-19 psychosocial impact on DE through psychological distress. | Low |
| Raykos et al. (2021) | Australia | Mixed methods | ED patients | 25 | Yes | 93.0% | 24.2 (7.6) | NS | 72% employed or studying | No | EDE-Q | No significant differences in the reduction of DE between the pre- and pandemic groups over treatment. | Med |
| Richardson et al. (2020) | Canada | Mixed methods | ED sample | NS | Yes | NS | NS | NS | NS | Children and adolescents | Self-reported symptom frequency | Significant increase in contacts to the National Eating Disorder Information Centre in 2020 compared to 2018, but not 2019. Significant increase in dieting/restriction, over-exercising, and purging in 2020 compared to 2018 and 2019. Four themes were identified: lack of access to treatment, worsening of symptoms, loss of control, and increased need for support. | Med |
| Author (date) | Country | Study Design | Study Population | Sample Size | ED Clinical Status | Gender Distribution (% Female) | Age Distribution (*M*(*SD*)) | Ethnicity / Race (% White) | SES | Minority / Understudied Group | ED Measure of Interest | Main Findings and Effect Size (if available) | MMAT |
| Scharmer et al. (2020) | United States | Cross-sectional | University students | 295 | No | 65.1% | 19.7 (2.0) | NS | NS | No | EDE-Q | EDE-Q scores were positively associated with COVID-19 anxiety. | Low |
| Schlegl, Maier, et al. (2020) | Germany | Cross-sectional | Former patients with AN | 159 | Yes | 100% | 22.4 (8.7) | NS | NS | No | Self-developed questionnaire assessing ED symptoms | Most participants agreed the pandemic worsened their ED symptoms and reported increased ED cognitions such as drive for thinness, fear of gaining weight, and eating, shape, and weight concerns. Majority disagreed the pandemic created new ED symptoms. | Med |
| Schlegl, Meule, et al. (2020) | Germany | Cross-sectional | Former patients with BN | 55 | Yes | 100% | 24.4 (6.4) | NS | NS | No | Self-developed questionnaire assessing ED symptoms | Most participants agreed the pandemic worsened their ED symptoms and reported increased ED cognitions, such as shape, eating, and weight concerns, fear of gaining weight, body dissatisfaction, and drive for thinness. Majority disagreed the pandemic created new ED symptoms. | High |
| Serin and Koç (2020) | Turkey | Cross-sectional | University students | 1,064 | No | 58.7% | NS | NS | NS | No | DEBQ | Participants who self-isolated had higher external eating than those who did not self-isolate. No significant differences in restricted eating between the self-isolate groups. | Med |
| Simone et al. (2021) | United States | Mixed methods | Young adults | 720 | No | 62.1% | 24.7 (2.0) | 29.6% | 32.7% of participants were classified as having low parental SES | No | Unhealthy weight control behaviours (UWCBs) and binge eating | Pandemic related stress management (B = 0.88, 95% CI 0.80, 0.97) was negatively associated with UWCBs. Pandemic related stress management (OR = 0.9, 95% CI 0.95, 1.27) was negatively associated with binge eating. Participants reported six main themes: mindless eating, increased and decreased food consumption, eating to cope, and increased DE. | High |
| Author (date) | Country | Study Design | Study Population | Sample Size | ED Clinical Status | Gender Distribution (% Female) | Age Distribution (*M*(*SD*)) | Ethnicity / Race (% White) | SES | Minority / Understudied Group | ED Measure of Interest | Main Findings and Effect Size (if available) | MMAT |
| Springall et al. (2021) | Australia | Retrospective chart review | Adolescent ED patients | 457 | Yes | 2017 = 80.6%, 2018 = 82.2%, 2019 = 90.2%, 2020 = 90.1% | 2017 = 14.9 (2.0), 2018 = 14.8 (1.9), 2019 = 15.1 (1.8), 2020 = 15.0 (1.8) | NS | NS | Children and adolescents | DSM-5 | Significant increase in ED presentations in 2020 compared to 2017 to 2019. In 2020, 40.4% of patients reported the onset of their ED behaviours coincided with the lockdown. | High |
| Stewart et al. (2021) | Canada | Longitudinal design | Adolescents accessing mental health services | 6,909 | No | Pre-pandemic = 54.9%, pandemic = 61.1% | Pre-pandemic = 15.2 (1.7), pandemic = 15.1 (1.7) | NS | Pre-pandemic = 24.5% high neighbourhood income quartile, pandemic = 25.2% high neighbourhood income quartile | Children and adolescents | NS | No significant differences in binge eating or fasting/major restrictions of diet in the last 30 days during pre-pandemic and pandemic times. | High |
| Termorshuizen et al. (2020) | Sweden | Cross-sectional | Individuals with self-reported EDs | United States = 511, Netherlands = 510 | Yes | United States = 95.0%, Netherlands = 98.0% | United States = 30.6 (9.4), Netherlands = NS | NS | NS | No | Questions about COVID-19-related concerns on ED’s. | Most participants were concerned about worsening of ED due to lack of structure, triggering environment, and lack of social support caused by the pandemic. | Med |
| Thompson and Bardone-Cone (2021) | United States | Cross-sectional | Postpartum women | 369 | No | 100% | Postpartum = 30.2 (4.2), control = 29.8 (4.3) | Postpartum = 92.7%, control = 83.2% | The control group had a significantly higher level of education. | No | EAT-26 | No significant differences in ED symptoms between groups during the pandemic. | High |
| Trott et al. (2021) | United Kingdom | Longitudinal design | Health club users | 319 | No | 84.0% | 36.8 (11.8) | NS | NS | No | EAT-26 | Significant increase in DE scores post-COVID-19 compared to pre-COVID-19 lockdown. | Low |
| Vaccaro et al. (2021) | Italy | Observational design | ED hospital admissions | NS | Yes | NS | NS | NS | NS | No | NS | 40.9% increase in new patients treated for ED’s in the first 6 months of 2020 compared to the same period in 2019. | Med |
| L. Wang et al. (2021) | China | Cross-sectional | Children and adolescents | 12,186 | No | 47.8% | 6-11 years = 9.1 (1.4), | NS | 55.5% had a parental education | Children and adolescents | Yes/No response to ED | EDs were a significant risk factor associated with physical and behavioural | High |
| Author (date) | Country | Study Design | Study Population | Sample Size | ED Clinical Status | Gender Distribution (% Female) | Age Distribution (*M*(*SD*)) | Ethnicity / Race (% White) | SES | Minority / Understudied Group | ED Measure of Interest | Main Findings and Effect Size (if available) | MMAT |
|  |  |  |  |  |  |  | 12-16 years = 13.9 (1.4) |  | status of 12 years or more |  |  | problems in Wuhan and outside Wuhan during the pandemic (OR = 2.71, 95% CI 2.35 to 3.11; *p* < .001). |  |
| S. D. Wang et al. (2021) | United States | Cross-sectional | Mothers | 197 | No | 100% | 37.6 (6.9) | 58.1% | 53.5% had completed a university degree of higher. | No | R-TFEQ | No significant relationship between COVID-19 stress and uncontrolled eating | Med |
| Zeiler et al. (2021) | Austria | Qualitative interview | Adolescent patients with anorexia nervosa and their parents | Patients = 13, parents = 10 | Yes | Patients = 100%, parents = 80.0% | Patients = 15.9 (1.4), parents = 49.7 (2.4) | NS | 70% of parents had a university degree | Children and adolescents | DSM-5 | Four main themes were identified from patients in related to the COVID-19 pandemic: restriction of personal freedom, interruption of treatment routine, changes in ED symptoms, and opportunities of the COVID-19 period. Three main themes were identified from parents in related to the COVID-19 pandemic: changes in daily routines, parents’ perspective of treatment, and challenges and benefits for ED symptoms and mental health of the child. | High |
| Zhou and Wade (2021) | Australia | Randomised controlled trial | University students | 100 | No | 100% | 19.9 (2.0) | 88.0% | NS | No | EDE-Q | Significant increase in weight concerns (*d* = 0.46, 95% CI 0.06, 0.87) and DE (*d* = 0.55, 95% CI 0.15, 0.96) between the pre-pandemic and pandemic groups. | Low |
| *Note.* ED = eating disorder, DE = disordered eating, SES = socioeconomic status, MMAT = Mixed Methods Appraisal Tool, , ELCS = Eating Loss of Control Scale, DSM = Diagnostic and Statistical Manual, of Mental Disorders, EDE-Q = Eating Disorder Examination-Questionnaire, BEDS = Binge-Eating Disorder Screener, CIA = Clinical Impairment Assessment, EDDS = Eating Disorder Diagnostic Scale, EDI = Eating Disorder Inventory, EAT = Eating Attitudes Test, TFEQ = Three Factor Eating Questionnaire, EPSI = Eating Pathology Symptoms Inventory, EDE = Eating Disorder Examination, K10 = Kessler Psychological Distress Scale, DEBQ = Dutch Eating Behaviour Questionnaire, SWED = Stanford-Washington University Eating Disorder Screener, WCS = Weight Concerns Scale, SEED = Short Evaluation of Eating Disorder, BES = Binge Eating Scale. | | | | | | | | | | | | | |
